# Nationwide outcomes of fenestrated endovascular aneurysm repair

**DOI:** 10.1093/bjs/znag037

**Published:** 2026-03-27

**Authors:** Aurélien M Guéroult, British Society of Endovascular Surgery and the GLOBALSTAR Collaborators, S Dindyal, V Gadhvi, M Hossain, A Kordzadeh, D J Adam, M Claridge, M Hook, K Powezka, M Vezzosi, S Hobbs, P Bevis, M Brooks, M Dewi, J Hardman, R Hinchliffe, G Ambler, A Awopetu, J R Boyle, C Cousins, P D Hayes, T Mehta, G Penney, T C See, K Varty, A Winterbottom, M Bown, E Choke, M McCarthy, A Saratzis, R Sayers, A Tambyraja, J A Brennan, R Canavati, R K Fisher, S Holder, R G McWilliams, J B Naik, S Vallabhaneni, A Alshiekh, F Farquharson, F Serracino-Inglott, R E Bell, A Burdess, M J Clarke, T El-Sayed, R Jackson, J McCaslin, S Nandhra, J D Rose, A Sharif, V Wealleans, R Williams, L Wilson, M G Wyatt, W Al-Jundi, I Aziz, C Knight, N Mohammed, P Stather, O Agu, C Bishop, D Boardley, J Constantinou, J Cross, M Davis, C Eng, R Gumama, J Hague, G Hamilton, P L Harris, K Ivancev, J Raja, T Richards, D Simring, Y Uddin, N Elzefzaf, G Gamtkitsulashvili, S Mathew, B Patterson, C Atkinson, B Azhar, J Budge, R Furlong, I Loftus, T Loosemore, R Morgan, A Pouncey, I Roy, M M Thompson, C Bicknell, P Bourke, N Cheshire, I Franklin, R Gibbs, M Hamady, A James, M P Jenkins, C Riga, S Salim, M Abdelhalim, S Abisi, S Black, T W Carrell, M Dialynas, P Gkoutzios, B Modarai, T Sabharwal, R Salter, R Sandford, M R Tyrrell, M Waltham, C J Wilkins

**Affiliations:** St George’s Vascular Institute, City St George’s University of London, London, UK; School of Health and Medical Sciences, City St George’s University of London, London, UK

## Abstract

**Background:**

Fenestrated endovascular aneurysm repair (FEVAR) is increasingly used in patients who are not eligible for standard endovascular aneurysm repair (EVAR) devices due to anatomical constraints, principally of the proximal aortic neck. The long-term outcomes of FEVAR are not well described and therefore the aim of this study was to perform an analysis of a national registry in the UK.

**Methods:**

The UK GLOBALSTAR registry (2003–2022) was analysed to report the long-term outcomes of FEVAR. Patients were included if they underwent FEVAR with custom-made devices for infradiaphragmatic aortic aneurysmal disease. Patients with extent I-III thoracoabdominal aneurysms or those who underwent other complex EVAR techniques were excluded. Time-to-event analyses were conducted for survival, aneurysm-related mortality, reintervention, endoleak, and target vessel patency. Subgroup analyses were conducted for octogenarians and patient sex.

**Results:**

Some 1651 patients across 15 centres were included. The median age was 75 (interquartile range 69–79) years and 87.9% were male. Estimated survival at 3, 5, and 10 years was 79.5% (95% c.i. 77.6% to 81.5%), 64.1% (95% c.i. 61.7% to 66.6%), and 30.5% (95% c.i. 27.7% to 33.5%) respectively; median survival was 6.9 (95% c.i. 6.6 to 7.2) years. Aneurysm-related mortality was 6.4% (95% c.i. 5.1% to 7.7%) at 10 years. There were 36 secondary sac ruptures over follow-up. The cumulative incidence of reintervention at 3, 5, and 7 years was 21.5% (95% c.i. 19.5% to 23.6%), 25.6% (95% c.i. 23.4% to 27.8%), and 27.6% (95% c.i. 25.3% to 30.0%) respectively. Graft-related complications accounted for 74.5% of reinterventions. Women had significantly worse 2-year survival (81.1% (95% c.i. 75.8% to 86.8%)) than men (86.1% (95% c.i. 84.3% to 87.9%)) (*P* = 0.041), driven by increased perioperative mortality. Beyond 2 years, the differences in survival were not statistically significant. Octogenarians had equivalent survival (89.4% (95% c.i. 86.4% to 92.4%)) to non-octogenarians (92.1% (95% c.i. 90.7% to 93.6%)) up to 1 year (*P* = 0.074). Octogenarians’ median survival was 5.4 (95% c.i. 5.0 to 6.1) years.

**Conclusions:**

Long-term outcomes of FEVAR appear acceptable, with low rates of secondary sac rupture. FEVAR is associated with a significant risk of reintervention, with graft-related complications being a key driver, suggesting effective planning and graft surveillance are key to long-term durability.

## Introduction

Complex endovascular repair is indicated in ∼30–40% of abdominal aortic aneurysms (AAAs) with short or hostile necks^1^. In this setting, fenestrated endovascular aneurysm repair (FEVAR) is recommended as a valid treatment option in clinical guidelines, but long-term durability continues to be questioned^2,3^.

Evaluating long-term outcomes of FEVAR is important following the UK-COMPASS study^4^, which reported inferior mid-term survival rates after FEVAR compared with open surgical repair (OSR) for all but one anatomical subgroup (≤4 mm necks) of juxtarenal AAAs^4^. In contrast, a recent single-centre study reported superior mid-term survival after FEVAR for those aged <70 years, suggesting patient selection is key^5^. FEVAR use is increasing worldwide^6^, with many patients favouring endovascular aneurysm repair (EVAR) over OSR due to lower procedural risk and shorter hospital stays^7^.

An RCT comparing OSR and FEVAR is not currently deliverable, as clinicians lack equipoise^8^. Clinicians and patients remain reliant on small studies or those with limited follow-up. Large registries such as the US Vascular Quality Initiative (VQI) have enabled short-term outcomes to be reported^9^, but are constrained by short-term follow-up^3^.

The GLOBALSTAR registry, established in 2006 by the British Society of Endovascular Therapy, is a UK registry of complex EVAR with uniquely long follow-up, offering an important opportunity to evaluate long-term FEVAR durability. The aim of this study was to report the long-term outcomes of FEVAR.

## Methods

This article conforms with the STROBE checklist for reporting cohort studies^10^.

### Study design

The UK GLOBALSTAR registry (2003–2022) was analysed to report the long-term outcomes of FEVAR.

### Ethics

Ethical approval for the registry and this associated research was renewed in November 2023 (23/NW/0327, IRAS: 326733).

### Inclusion and exclusion criteria

Inclusion criteria were: adults, infradiaphragmatic aneurysmal disease irrespective of anatomy, custom-made FEVAR alone, and operated on during years 2003–2022. Thoracoabdominal aortic aneurysms (extent I–III), non-aneurysmal disease (aortic dissection), and non-FEVAR complex EVAR techniques (branched EVAR, chimney EVAR, and physician-modified endografts) were excluded. Local databases for complex EVAR were screened and patients were included in accordance with the inclusion/exclusion criteria.

### Variables

Outcomes were categorized as primary and secondary to reflect clinical importance and pre-specified priority of reporting. The primary outcome was all-cause mortality. Secondary outcomes included: reintervention, aneurysm-related mortality, secondary sac rupture, endoleak incidence, and target vessel patency (TVP; stented visceral branches of the abdominal aorta). These outcomes were selected after conducting a systematic review and meta-analysis of the literature^3^ and ascertainment of patient priorities for reporting via the City St George’s vascular patient advisory group. Aneurysm-related mortality was defined as death during the index admission, death within 30 days of the procedure or reintervention, death due to procedural complications, and death due to secondary sac rupture. TVP was defined as visceral stent occlusion and numbers at risk correspond to individuals rather than target vessels, for more meaningful interpretation.

Full details of the preoperative, intraoperative, and postoperative variables that were collected are available in the study data collection protocol provided (see the *Supplementary material*). Definitions and diagnostic criteria were in accordance with published reporting standards^11^. Aneurysm sac size change was calculated as the difference between the preoperative anteroposterior (AP) sac diameter and the latest AP sac diameter measurement during follow-up, with regressing sacs defined as having at least a −5 mm change in sac diameter, stable sacs defined as having a ±<5 mm change in sac diameter, and expanding sacs defined as having at least a +5 mm change in sac diameter^11^.

### Data sources

Follow-up for all patients was carried out by local collaborators through retrospective review of electronic patient records (EPRs) for inpatient and outpatient episodes, using the standardized GLOBALSTAR data collection protocol. CT and duplex ultrasonography were used during follow-up as part of local surveillance practices; imaging reports were reviewed to collect TVP and endoleak data. The primary outcome data were obtained by cross-referencing EPRs with the National Health Service (NHS)-wide mortality database (Spine, NHS Digital) derived from death records from the Office for National Statistics; this was performed locally in each centre.

### Study size

The retrospective cohort design and descriptive nature of the study obviated the requirement for traditional power calculations or formal adjustment for multiple comparisons. Consequently, outcome estimates are intended to be interpreted descriptively and no confirmatory hypothesis testing is implied.

### Statistical methods

Statistical analyses were conducted using R (version 4.5.0)^12^; full statistical methods are available in the *Supplementary material*.

#### Statistical analyses

Descriptive statistics were used to report demographic, operative, and postoperative complication data.

Kaplan–Meier time-to-event analysis was conducted for survival. Cumulative incidence analyses with all-cause mortality treated as a competing risk^13^ were applied to reintervention, aneurysm-related mortality, endoleak incidence, and TVP. This approach was chosen to estimate crude, real-world event probabilities, acknowledging that death precludes the occurrence of these outcomes. This technique avoids overestimation of event-free outcome probabilities (hypothetical net risks) that would arise from treating death as non-informative censoring, as assumed in Kaplan–Meier analyses. A 10% threshold for data maturity (risk-set-based) was applied to time-to-event analyses; estimates were reported only up to time points at which 10% of the study population remained at risk for the event of interest, to avoid unreliable estimates based on very small risk sets, as recommended by Pocock *et al*.^14^. Log rank subgroup analyses were applied to compare sex and age-based subgroups.

#### Quantitative variables

In time-to-event analyses, age was dichotomized to compare octogenarian and non-octogenarian subgroups^15,16^.

#### Strategy for missing data and loss to follow-up

Loss to follow-up was accounted for by censoring in time-to-event analyses. Missingness involved complete case analysis with listwise deletion. For cases where >5% of data were missing, missingness was assumed as conditional of the covariates available in the data and this was dealt with by multiple imputation using chained equations.

#### Sensitivity analyses

Sensitivity analyses were performed by modelling preoperative variables against survival at 1 year and 5 years; logistic regression was selected as the best performing model by discrimination and calibration. These analyses were conducted as exploratory sensitivity analyses to compare survival between sex and octogenarian subgroups. They were not intended to support causal inference or to develop a formal prediction model, and results should be interpreted descriptively as group-level survival comparisons only. Due to GLOBALSTAR’s extended study interval, case recency was identified as a potential key confounder. Chronologically stratified subgroup analyses were performed for survival, reintervention, and endoleak incidence as further sensitivity analyses.

## Results

### Participants

Screening of the GLOBALSTAR registry identified 2153 patients, of whom some 1651 patients met the inclusion criteria (*Fig. S1* and *Table S1*).

### Cohort characteristics

The median age of included patients was 75 (interquartile range (i.q.r.) 69–79) years and 87.9% were male (*Table 1*). A differentiation is made between follow-up for all-cause mortality (checked using local software connected to NHS Spine) and follow-up with surveillance imaging. The cohort’s total accrued follow-up for all-cause mortality was 9142.1 person-years (s.d. = 3.5) and the cohort’s total accrued follow-up with surveillance imaging was 6018.9 person-years (s.d. = 7.7).

**Table 1:** Preoperative, operative, and postoperative data

Variables	Values	Percentage of missing data (denominator)
**Preoperative data**		
Age at operation (years), median (i.q.r.)	75 (69–79)	0.0 (1651)
Octogenarians	24.5 (404)	0.0 (1651)
Male	87.9 (1433)	1.3 (1630)
Year of operation, median (i.q.r.)	2016 (2012–2018)	0.0 (1651)
Mortality follow-up (years), median (i.q.r.)	5.2 (2.8–7.6)	0.0 (1651)
Imaging follow-up (years), median (i.q.r.)	3.3 (1.0–6.1)	0.0 (1651)
Aneurysm diameter (mm), median (i.q.r.)	62 (58–69)	4.8 (1571)
ASA grade, median (i.q.r.)	III (III–III)	34.5 (1081)
Co-morbidities		
Diabetes	15.5 (251)	1.6 (1624)
Ischaemic heart disease	44.7 (727)	1.5 (1626)
Heart failure	8.7 (142)	1.6 (1624)
Hypertension	73.7 (1197)	1.6 (1624)
Chronic kidney disease	16.1 (261)	1.8 (1621)
Smoker or ex-smoker	75.4 (1163)	6.6 (1542)
Peripheral arterial disease	11.7 (190)	1.9 (1621)
Prior aortic surgery	12.9 (175)	18.1 (1352)
Preoperative blood results		
Haemoglobin (g/l), median (i.q.r.)	138 (126–149)	17.9 (1356)
Sodium (mmol/l), median (i.q.r.)	139 (137–141)	18.4 (1348)
Potassium (mmol/l), median (i.q.r.)	4.4 (4.1–4.7)	18.5 (1345)
Urea (mmol/l), median (i.q.r.)	6.4 (5.3–7.9)	28.0 (1189)
Creatinine (μmol/l), median (i.q.r.)	94 (79–117)	15.2 (1400)
**Operative data**		
Graft characteristics		
Number of fenestrations, median (i.q.r.)	3 (2–4)	2.5 (1609)
Manufacturers		
Cook Zenith	86.1 (1382)	7.8 (1521)
Terumo Anaconda	6.9 (110)	7.8 (1521)
JOTEC	0.2 (4)	7.8 (1521)
Configurations		
Scallop only	1.2 (19)	2.5 (1609)
Single fenestration only	1.9 (31)	2.5 (1609)
Single fenestration + scallop	1.5 (24)	2.5 (1609)
Two fenestrations only	11.4 (183)	2.5 (1609)
Two fenestrations + scallop	11.2 (181)	2.5 (1609)
Three fenestrations	22.5 (363)	2.5 (1609)
Three fenestrations + scallop	11.8 (190)	2.5 (1609)
Four fenestrations	38.2 (616)	2.5 (1609)
Five fenestrations (inferior mesenteric artery or accessory renal arteries)	0.2 (3)	2.5 (1609)
Target vessels incorporated in repair		
Coeliac axis	16.4 (833)	2.5 (5068)
Superior mesenteric artery	26.3 (1335)	2.5 (5068)
Renal arteries	57.2 (2898)	2.5 (5068)
Inferior mesenteric artery	0.04 (2)	2.5 (5068)
**Postoperative data**		
Length of hospital stay (days), median (i.q.r.)	5 (3–8)	4.4 (1579)
Length of ITU stay (days), median (i.q.r.)	1 (0–2)	10.7 (1475)
Postoperative complications (individuals)	40.5 (629)	5.8 (1555)
Clavien–Dindo classification		
Minor complications (individuals)	27.1 (421)	5.8 (1555)
Grade I	16.5 (256)	5.8 (1555)
Grade II	10.6 (165)	5.8 (1555)
Major complications (individuals)	13.2 (205)	5.8 (1555)
Grade III	5.2 (81)	5.8 (1555)
Grade IV	5.4 (84)	5.8 (1555)
Grade V	2.6 (40)	5.8 (1555)
Cardiovascular (events)	3.5 (85)	5.8 (1555)
MACEs	3.5 (54)	5.8 (1555)
Cardiac ischaemia (MI or ACS)	1.8 (28)	5.8 (1555)
Cardiac arrest	0.5 (8)	5.8 (1555)
Pulmonary embolism	0.1 (2)	5.8 (1555)
Aortic rupture	0.1 (1)	5.8 (1555)
Type B aortic dissection	0.3 (4)	5.8 (1555)
Stroke	0.8 (13)	5.8 (1555)
Cholesterol embolization	0.1 (1)	5.8 (1555)
Other	1.8 (28)	5.8 (1555)
Arrhythmia	1.6 (25)	5.8 (1555)
Exacerbation of heart failure	0.2 (3)	5.8 (1555)
Lower limb (events)	5.3 (82)	5.8 (1555)
Acute limb ischaemia	1.3 (20)	5.8 (1555)
Claudication	0.5 (8)	5.8 (1555)
Access vessel haemorrhage	0.8 (13)	5.8 (1555)
Access vessel dissection/pseudoaneurysm	0.5 (7)	5.8 (1555)
Minor groin complications*	2.2 (34)	5.8 (1555)
Neurological (events)	2.7 (40)	5.8 (1555)
Permanent complete spinal cord ischaemia, paraplegia (spinal cord ischaemia grade 3)	0.5 (8)	5.8 (1555)
Permanent incomplete spinal cord ischaemia (spinal cord ischaemia grade 2)	0.4 (6)	5.8 (1555)
Transient spinal cord ischaemia with complete resolution (spinal cord ischaemia grade 1)	0.8 (12)	5.8 (1555)
Transient ischaemic attack	0.2 (3)	5.8 (1555)
Seizure	0.1 (1)	5.8 (1555)
Delirium	0.7 (11)	5.8 (1555)
Gastrointestinal (events)	3.1 (48)	5.8 (1555)
Ischaemic complications	1.4 (21)	5.8 (1555)
Bowel ischaemia	0.9 (14)	5.8 (1555)
Gastric ischaemia	0.1 (1)	5.8 (1555)
Splenic ischaemia	0.3 (4)	5.8 (1555)
Ischaemic cholecystitis	0.1 (2)	5.8 (1555)
Other	1.7 (27)	5.8 (1555)
Gastrointestinal bleed	0.5 (8)	5.8 (1555)
Ileus	0.8 (13)	5.8 (1555)
*Clostridioides difficile* and non-ischaemic colitis	0.3 (5)	5.8 (1555)
Pancreatitis	0.1 (1)	5.8 (1555)
Renal† (events)	5.2 (81)	5.8 (1555)
Dialysis or haemofiltration	1.0 (16)	5.8 (1555)
AKI	20.6 (321)	5.8 (1555)
Stage 1	15.7 (244)	5.8 (1555)
Stage 2	3.0 (46)	5.8 (1555)
Stage 3	2.0 (31)	5.8 (1555)
Postoperative creatinine change (µmol/l), median (i.q.r.)	+9 (−1 to +27)	46.3 (887)
Renal haemorrhage	1.3 (20)	5.8 (1555)
Renal infarct	2.3 (35)	5.8 (1555)
Hydronephrosis	0.1 (2)	5.8 (1555)
Minor urological complications‡	1.2 (19)	5.8 (1555)
Respiratory (events)	3.8 (59)	5.8 (1555)
Respiratory failure/reintubation	0.9 (14)	5.8 (1555)
Pulmonary oedema	0.3 (5)	5.8 (1555)
Pneumonia	2.6 (40)	5.8 (1555)
Other systemic complications (events)	2.0 (31)	5.8 (1555)
SIRS, sepsis, and MOF	1.3 (20)	5.8 (1555)
PIS and PUO	1.0 (15)	5.8 (1555)
Sac size change		
Sac size change (mm), median (i.q.r.)	−6 (−16 to +2)	11.8 (1400)
Measurement time (years), median (i.q.r.)	3.1 (1.0–5.9)	11.8 (1400)
Sac behaviour subgroups		
Sac regression	54.6 (765)	11.8 (1400)
Stable sac	27.1 (379)	11.8 (1400)
Sac expansion	18.3 (256)	11.8 (1400)

Values are % (*n*) unless otherwise indicated. *Minor bleed, haematoma, seroma, lymph leak, infection, and wound dehiscence. †Three patients suffered more than one MACE. ‡Urinary retention, urinary tract infection, traumatic catheter insertion, and transient haematuria. §Excludes AKI and minor urological complications. i.q.r., interquartile range; ITU, intensive treatment unit; MACEs, major adverse cardiovascular events; MI, myocardial infarction; ACS, acute coronary syndrome; AKI, acute kidney injury; SIRS, systemic inflammatory response syndrome; MOF, multiorgan failure; PIS, post-implantation syndrome; PUO, pyrexia of unknown origin.

### Early outcomes

Major perioperative morbidity (Clavien–Dindo grades III–V)^17^ was observed in 13.2% of the cohort, including 2.6% in-hospital mortality. This included 3.5% major adverse cardiovascular events (MACEs) and 0.5% permanent complete spinal cord ischaemia (paraplegia; European Society for Vascular Surgery spinal cord ischaemia grade 3)^2^. Acute kidney injury (AKI) according to the Risk, Injury, Failure, Loss of kidney function, and End-stage kidney disease (RIFLE) criteria was common (20.6%), but mostly stage 1 (76.0% of AKIs), with a postoperative change in creatinine of +9 (i.q.r. −1 to +27) µmol/l, and rarely translated into renal replacement therapy (1.0%), with 0.8% permanent dialysis and 0.2% acute haemofiltration.

The median sac size change was −6 (i.q.r. −16 to +2) mm, with a median measurement time of 3.1 (i.q.r. 1.0–5.9) years. At latest imaging, 51.6% of individuals were found to have sac regression, with a median change of −14 (i.q.r. −23 to −9) mm, and 17.7% of individuals were found to have sac expansion, with a median change of +12 (i.q.r. +7 to +22) mm.

### Long-term outcomes

#### Survival

**Fig. 1:**
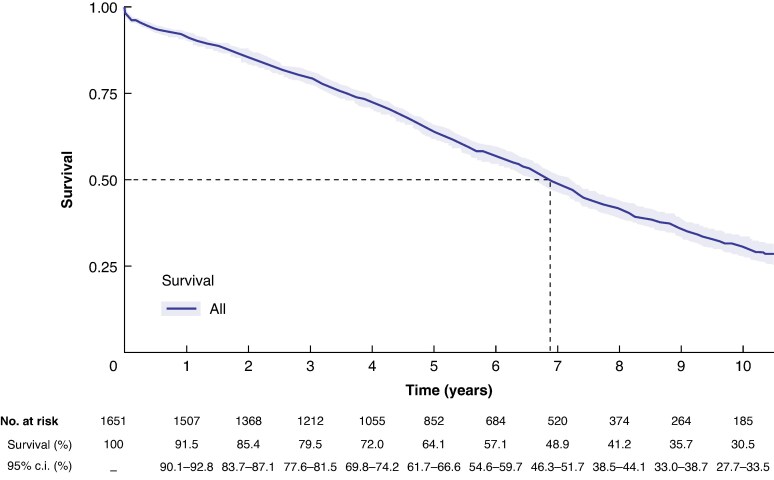
Survival: Kaplan–Meier time-to-event analysis Survival (solid line) with 95% c.i. ribbon. The dashed line indicates the median survival.

The median follow-up was 5.2 (i.q.r. 2.8–7.5) years. There were 968 deaths during follow-up (*Fig. 1*). Data maturity, using a 10% threshold, reached 10 years (185 patients at risk at 10 years). Survival at 1, 3, 5, and 10 years was 91.5% (95% c.i. 90.1% to 92.8%), 79.5% (95% c.i. 77.6% to 81.5%), 64.1% (95% c.i. 61.7% to 66.6%), and 30.5% (95% c.i. 27.7% to 33.5%). The median survival was 6.9 (95% c.i. 6.6–7.2) years.

#### Aneurysm-related mortality and secondary sac rupture

**Fig. 2:**
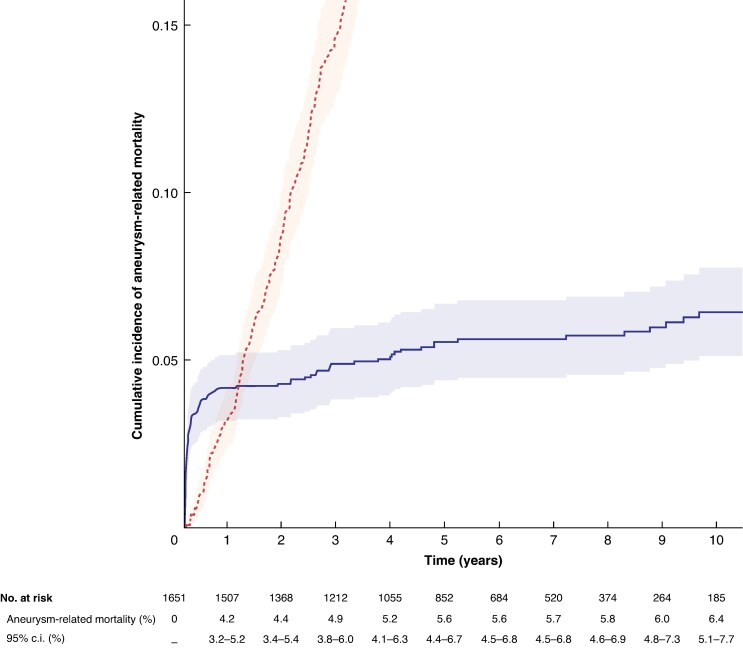
Cumulative incidence of aneurysm-related mortality with all-cause mortality as a competing risk Aneurysm-related mortality (solid line) and all-cause mortality as a competing risk (dashed line) with 95% c.i. ribbons.

The cumulative incidence of aneurysm-related mortality with all-cause mortality as a competing risk is presented in *Fig. 2*. Data maturity, using a 10% threshold, reached 10 years (185 patients at risk at 10 years). The cumulative incidence of aneurysm-related mortality at 1, 3, 5, and 10 years was 4.2% (95% c.i. 3.2% to 5.2%), 4.9% (95% c.i. 3.8% to 6.0%), 5.6% (95% c.i. 4.4% to 6.7%), and 6.4% (95% c.i. 5.1% to 7.7%). There were 104 aneurysm-related deaths during follow-up: 49 deaths within 30 days of index FEVAR (40 in-hospital deaths) and 55 aneurysm-related deaths during follow-up beyond 30 days, including 15 deaths within 6 months due to procedural morbidity (such as end-stage renal failure) and 40 deaths across the whole follow-up interval due to graft-related complications (within 30 days of reintervention, graft infection, or secondary sac rupture).

There were 36 secondary sac ruptures captured over the follow-up interval (2.2%); 19 (52.8%) leading to death and 17 (47.2%) leading to survival after successful reintervention. There were 3 rupture events (8.3%) within 6 months of FEVAR, 10 rupture events (27.8%) beyond 6 months to 4 years, 13 rupture events (36.1%) at >4–8 years, and 10 rupture events (27.8%) beyond 8 years. The incidence of secondary sac rupture was calculated as 3.9 events per 1000 person-years. The majority of secondary sac ruptures were driven by endoleaks (30 of 36 (83.3%)). Endoleaks detected preceding rupture were 36.7% type 1a/1b, 50.0% type 3, and 13.3% type 2 or undetermined. Almost half of patients with an endoleak-related rupture (13 of 30 (43.3%)) underwent a reintervention targeting the endoleak before rupture: three reinterventions for a type 1a/1b endoleak, two reinterventions for a type 2 endoleak, and eight reinterventions for a type 3 endoleak.

#### Reintervention

**Fig. 3:**
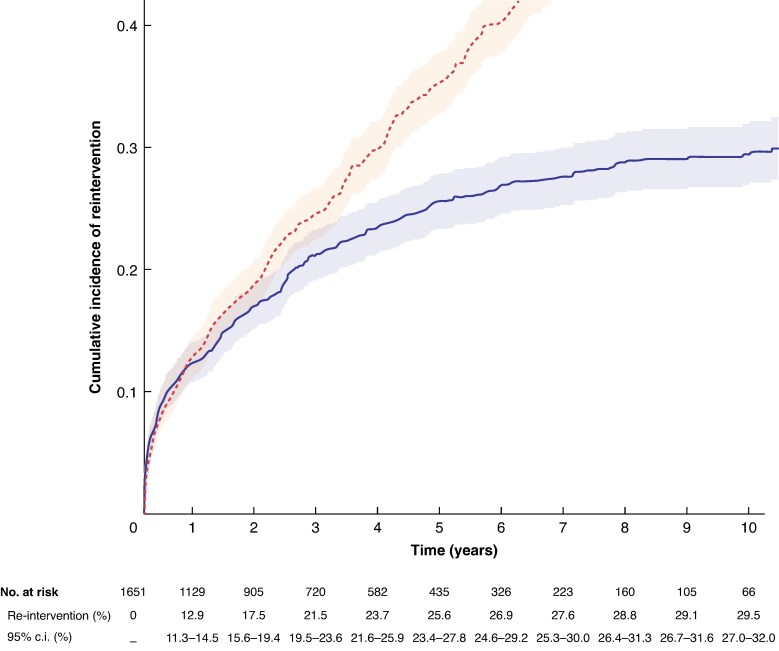
Cumulative incidence of reintervention with all-cause mortality as a competing risk Reintervention (solid line) and all-cause mortality as a competing risk (dashed line) with 95% c.i. ribbons. Shading in the number at risk table indicates years after data maturity.

Data maturity, using a 10% threshold, reached 7 years (223 patients at risk at 7 years). The cumulative incidence of reintervention at 1, 3, 5, and 7 years was 12.9% (95% c.i. 11.3% to 14.5%), 21.5% (95% c.i. 19.5% to 23.6%), 25.6% (95% c.i. 23.4% to 27.8%), and 27.6% (95% c.i. 25.3% to 30.0%) (*Fig. 3*). Reintervention affected 423 individuals (25.6%) and more than one reintervention was performed on 111 individuals (6.7%). Endovascular procedures accounted for 87.5% of first reinterventions, with the main driver for reintervention being graft-related indications (74.5%); there were near equivalent proportions of reintervention for type 3 endoleak (20.6%), stenosis/thrombosis/occlusion of the main body or iliac limbs (18.4%), and stenosis/thrombosis/occlusion of visceral stents (19.4%). The median time to first reintervention (excluding reinterventions within 30 days) was 1.7 (i.q.r. 0.6–3.2) years (*Table 2*).

**Table 2:** First reintervention indications and procedural details (*n* = 1651)

	Values
Any reintervention	25.6 (423)
>1 reintervention	6.7 (111)
**Timing of reintervention**	
Perioperative reinterventions (up to 30 days)	23.6 (100)
>30 days to 6 months	17.5 (74)
>6 months to 4 years	44.4 (188)
>4–8 years	12.5 (53)
>8 years	1.9 (8)
Indications for first reintervention*	
Graft-related indications	
Any	74.5 (315)
Stenosis/thrombosis/occlusion, main body or iliac limbs	18.4 (78)
Stenosis/thrombosis/occlusion, visceral stents	19.4 (82)
Type 1a or 1b endoleak	8.7 (37)
Type 3 endoleak	20.6 (87)
Migration	2.8 (12)
Graft infection	0.2 (1)
Proximal aortic dilatation	2.4 (10)
Secondary rupture	1.9 (8)
Non-graft-related vascular indications	
Any	20.1 (85)
Type 2 endoleak	13.7 (58)
Sac expansion	2.6 (11)
Native vessel stenosis/thrombosis/occlusion	2.6 (11)
Native vessel aneurysm	1.2 (5)
Systemic indications	
Any	6.1 (26)
Renal failure	0.2 (1)
Severe abdominal pain	0.7 (3)
Bleeding	3.3 (14)
Bowel ischaemia	1.4 (6)
Groin haematoma or infection	0.5 (2)
**First reintervention procedures**	
Endovascular†	87.5 (370)
Proximal extension/stabilization‡	3.5 (15)
Main body relining	2.6 (11)
Iliac limb angioplasty, relining, or extension	24.1 (102)
Visceral stent angioplasty or relining§	34.8 (147)
Diagnostic aortic angiogram	6.6 (28)
Type 1b endoleak embolization	0.5 (2)
Type 2 endoleak embolization	9.0 (38)
Internal iliac exclusion	2.1 (9)
Embolization of type 3 endoleak	1.7 (7)
Embolization of bleeding point	1.4 (6)
Native vessel angioplasty or stenting	1.2 (5)
Open	11.3 (48)
Bypass	2.4 (10)
Cross-over graft	2.4 (10)
Graft explantation	0.2 (1)
Native vessel endarterectomy or patch plasty	2.1 (9)
Groin exploration/haematoma evacuation	1.7 (7)
Laparotomy	2.4 (10)
Open secondary sac rupture repair	0.2 (1)
Unknown modality	
Secondary sac rupture repair	0.9 (4)

Values are % (*n*). *Three patients with more than one indication. †Includes eight endovascular reinterventions for secondary sac rupture, presented as the procedure performed. ‡One endoanchor procedure. §One suction thrombectomy and lysis to both renals and coeliac.

#### Endoleak

Data maturity, using a 10% threshold, reached 7 years (196 patients at risk at 7 years). At 1 year and 5 years, the cumulative incidence of type 1a/1b endoleak was 3.5% (95% c.i. 2.6% to 4.4%) and 5.6% (95% c.i. 4.4% to 6.7%) respectively, of type 2 endoleak was 16.2% (95% c.i. 14.4% to 18.0%) and 19.7% (95% c.i. 17.7% to 21.7%) respectively, and of type 3 endoleak was 5.8% (95% c.i. 4.7% to 7.0%) and 7.3% (95% c.i. 6.0% to 8.6%) respectively (*Fig. S2*). Endoleaks were detected in 559 individuals (33.9%) over the length of follow-up and 205 underwent reintervention for an endoleak-related indication (36.7%). For type 1a/1b endoleaks, 61 of 100 individuals (61.0%) underwent reintervention; for type 3 endoleaks and type 2 endoleaks, 84 of 128 individuals (65.6%) and 57 of 325 individuals (17.5%) underwent reintervention respectively.

#### TVP

Data maturity, using a 10% threshold, reached 8 years (287 patients at risk at 8 years). The cumulative incidence of loss of TVP at 1, 3, 5, and 8 years was 3.6% (95% c.i. 2.7% to 4.5%), 4.8% (95% c.i. 3.7% to 5.9%), 6.0% (95% c.i. 4.8% to 7.2%), and 7.0% (95% c.i. 5.6% to 8.4%). For the 103 patients who experienced loss of TVP, 90 patients (87.4%) lost patency of at least one renal artery, 11 patients (10.7%) lost patency of the superior mesenteric artery, and 2 patients (1.9%) lost coeliac patency (*Fig. S3*).

#### Survival subgroup analyses for sex and octogenarians

Perioperative mortality for women was 5.6% *versus* 2.6% for men. Subgroup log rank analysis for survival by sex demonstrated that the survival curves only converge at 3 years, with statistically significant differences in survival between the sexes at 1 year and 2 years (*P* = 0.002 and *P* = 0.041) (*Fig. S4*). Subgroup log rank analysis for survival by sex over the full length of follow-up is presented in *Fig. S4*, demonstrating no statistically significant difference in long-term survival (*P* = 0.564).

There was no statistically significant difference in early mortality for octogenarians up to 1 year (*P* = 0.074): non-octogenarians 92.1% (95% c.i. 90.7% to 93.6%) *versus* octogenarians 89.4% (95% c.i. 86.4% to 92.4%). Understandably, beyond 1 year the survival curves diverge (*Fig. S5*). The median survival time for octogenarians was 5.4 (95% c.i. 5.0 to 6.1) years *versus* 7.4 (95% c.i. 7.2 to 7.8) years for younger individuals.

#### Sensitivity analyses

Forest plots for multivariable logistic regression analyses examining 1-year and 5-year survival are presented in *Fig. S6* and *Fig. S7* respectively. In these exploratory analyses, female sex was associated with higher odds of 1-year mortality (OR 2.01 (95% c.i. 1.16 to 3.35)), whereas no clear association was observed with age.

Chronologically stratified survival analysis demonstrated statistically significant improved 4-year survival for cases performed after 2019 compared with 2003–2008 (paired log rank *P* = 0.016), but overall log rank across time intervals was not statistically significant (*P* = 0.258) (*Fig. S8*).

Chronologically stratified cumulative incidence of reintervention time-stamped at 4 years demonstrated no statistically significant difference between time intervals (Gray’s test *P* = 0.232). There was no statistical difference in cumulative incidence at 4 years across time intervals for type 1 endoleaks (*P* = 0.064) and type 3 endoleaks (0.751). There was a statistically significant difference in the cumulative incidence of type 2 endoleaks across time intervals (*P* = 0.047) (*Fig. S9*).

## Discussion

Demographic data reflect the real-world context for the current UK use of FEVAR in an elderly and co-morbid population. FEVAR does carry a significant burden of perioperative morbidity (major complication rate of 13.2%) though the rate of paraplegia was low (0.5%), consistent with limited aortic coverage. Despite this challenging context, hospital and intensive treatment unit (ITU) stays were short (median = 5 days and 1 day respectively). This may suggest short-stay care models for FEVAR are feasible, which would enhance the financial viability of FEVAR, especially if manufacturers reduced device costs. Driving down costs should be possible with greater case volumes being performed, more off-the-shelf solutions, advances in three-dimensional (3D)-printing manufacturing, increased market competition, and system-wide purchasing strategies with manufacturers.

This study is unique in presenting mature long-term outcomes for a large real-world cohort of FEVAR.

This study demonstrated acceptable long-term survival for FEVAR, consistent with a previous meta-analysis (1-year survival of 91.5% in this study *versus* 91.6%^3^ and 5-year survival of 64.1% in this study *versus* 65.1%^3^). Secondary sac rupture was a rare event (3.9 events per 1000 person-years); the higher incidence of late secondary sac rupture (>8 years) observed in the EVAR-1 trial’s long-term results for infrarenal EVAR was not replicated^18^. This is a key finding, which may suggest greater long-term durability for FEVAR compared with infrarenal EVAR, potentially due to more extensive proximal coverage and consequently more effective sealing.

The freedom from reintervention reported in this study is consistent with that reported in a previous meta-analysis (87.1% in this study *versus* 90.2%^3^ at 1 year and 74.4% in this study *versus* 73.8%^3^ at 5 years). Reintervention clearly remains a significant issue for FEVAR. Graft-related indications accounted for the majority of reinterventions (74.5%), with the main drivers being graft-related endoleaks (29.3%); stenosis/thrombosis/occlusion of visceral stents (19.4%) and stenosis/thrombosis/occlusion of the main body or iliac limbs (18.4%), which occurred in similar proportions. Optimizing device selection, procedural technique, homogeneous postoperative medical therapy, and surveillance to limit the impact of graft-related complications should be key targets for quality improvement in FEVAR. Further, the incidence of 7.0% of individuals losing TVP over follow-up may suggest that a surveillance strategy based solely on duplex ultrasonography focusing on the aneurysm sac is insufficient to detect early stenosis/thrombosis for timely reintervention.

Sex differences in perioperative survival were consistent with the literature, which reports worse outcomes for women after AAA repair^19^. There are several possible explanations for this observation, including more hostile access vessels and occult ischaemic heart disease. Full evaluation of sex-specific differences in this cohort will be a topic of future research.

Equivalent short-term survival for octogenarians compared with younger individuals suggests appropriate and effective patient selection. This may reflect the multidisciplinary approach to preoperative assessment for aortic surgery. The reported median survival of >5 years may justify the use of FEVAR for carefully selected octogenarian patients.

GLOBALSTAR results are generalizable in that the population is highly comparable in terms of age and co-morbidity to FEVAR cohorts reported in the literature^3^.

GLOBALSTAR is limited by its retrospective design and associated biases. Centres variably contributed cases after an enforced pause in data collection in 2020 due to the COVID-19 pandemic (date ranges in *Table S1*).

The study interval (2003–2022) encompasses changes in practice and technology: increased prevalence of more complex graft designs, percutaneous access and evolving delivery systems, bridging stent strategies, and procedural refinements with increasing institutional experience. Time-to-event analyses do not account for these factors. Sensitivity analyses (see the *Supplementary material*) showed statistically significant improved survival at 4 years in the most recent era (2020–2022) compared to the earliest era (2003-2008), potentially reflecting improved patient selection, improved devices, and more intensive surveillance.

Aneurysm morphology was not captured, so hostile anatomy was not accounted for. This is an important limitation, as anatomical complexity is a recognized determinant of both early and late outcomes of FEVAR and residual confounding cannot be excluded. However, this study was restricted to custom-made FEVAR for infradiaphragmatic AAAs, excluding branched configurations and extent I–III thoracoabdominal aneurysms, which carry greater anatomical variability and risk.

Despite these limitations, GLOBALSTAR presents acceptable long-term outcomes of FEVAR with good data maturity. FEVAR is associated with risk, but, with effective patient selection, offers an acceptable treatment option for complex AAA patients, with low rates of secondary rupture. Reinterventions were mainly graft-related, highlighting areas for quality improvement in device planning and surveillance.

In the absence of a randomized comparison with OSR, large cohorts such as GLOBALSTAR offer invaluable insights. A robust prospective complex EVAR registry with reliable data capture and validation is vitally needed. GLOBALSTAR is unique in terms of its real-world, unselected cohort and the length of its follow-up. It will inform shared decision-making between patients and clinicians in the treatment of complex AAAs.

## Supplementary Material

znag037_Supplementary_Data

## Data Availability

Data are currently embargoed for exclusive use of the GLOBALSTAR steering group; but, after the embargo, the data will become available for research purposes upon request and after review by the steering group.

## Collaborators

S. Dindyal (Basildon Hospital, Basildon, UK); V. Gadhvi (Basildon Hospital, Basildon, UK); M. Hossain (Basildon Hospital, Basildon, UK); A. Kordzadeh (Basildon Hospital, Basildon, UK); D. J. Adam (Heartlands Hospital, Birmingham, UK); M. Claridge (Heartlands Hospital, Birmingham, UK); M. Hook (Heartlands Hospital, Birmingham, UK); K. Powezka (Heartlands Hospital, Birmingham, UK); M. Vezzosi (Heartlands Hospital, Birmingham, UK); S. Hobbs (New Cross Hospital, Wolverhampton, UK); P. Bevis (Southmead Hospital, Bristol, UK); M. Brooks (Southmead Hospital, Bristol, UK); M. Dewi (Southmead Hospital, Bristol, UK); J. Hardman (Royal United Hospital, Bath, UK); R. Hinchliffe (Southmead Hospital, Bristol, UK); G. Ambler (Addenbrooke’s Hospital, Cambridge, UK); A. Awopetu (Addenbrooke’s Hospital, Cambridge, UK); J. R. Boyle (Addenbrooke’s Hospital, Cambridge, UK); C. Cousins (Addenbrooke’s Hospital, Cambridge, UK); P. D. Hayes (Addenbrooke’s Hospital, Cambridge, UK); T. Mehta (Addenbrooke’s Hospital, Cambridge, UK); G. Penney (Addenbrooke’s Hospital, Cambridge, UK); T. C. See (Addenbrooke’s Hospital, Cambridge, UK); K. Varty (Addenbrooke’s Hospital, Cambridge, UK); A. Winterbottom (Addenbrooke’s Hospital, Cambridge, UK); M. Bown (Leicester Royal Infirmary, Leicester, UK); E. Choke (Leicester Royal Infirmary, Leicester, UK); M. McCarthy (Leicester Royal Infirmary, Leicester, UK); A. Saratzis (Leicester Royal Infirmary, Leicester, UK); R. Sayers (Leicester Royal Infirmary, Leicester, UK); A. Tambyraja (Leicester Royal Infirmary, Leicester, UK); J. A. Brennan (Royal Liverpool University Hospital, Liverpool, UK); R. Canavati (Royal Liverpool University Hospital, Liverpool, UK); R. K. Fisher (Royal Liverpool University Hospital, Liverpool, UK); S. Holder (Royal Liverpool University Hospital, Liverpool, UK); R. G. McWilliams (Royal Liverpool University Hospital, Liverpool, UK); J. B. Naik (Royal Liverpool University Hospital, Liverpool, UK); S. Vallabhaneni (Royal Liverpool University Hospital, Liverpool, UK); A. Alshiekh (Manchester Royal Infirmary, Manchester, UK); F. Farquharson (Manchester Royal Infirmary, Manchester, UK); F. Serracino-Inglott (Manchester Royal Infirmary, Manchester, UK); R. E. Bell (Freeman Hospital, Newcastle upon Tyne, UK); A. Burdess (Freeman Hospital, Newcastle upon Tyne, UK); M. J. Clarke (Freeman Hospital, Newcastle upon Tyne, UK); T. El-Sayed (Freeman Hospital, Newcastle upon Tyne, UK); R. Jackson (Freeman Hospital, Newcastle upon Tyne, UK); J. McCaslin (Freeman Hospital, Newcastle upon Tyne, UK); S. Nandhra (Freeman Hospital, Newcastle upon Tyne, UK); J. D. Rose (Freeman Hospital, Newcastle upon Tyne, UK); A. Sharif (Freeman Hospital, Newcastle upon Tyne, UK); V. Wealleans (Freeman Hospital, Newcastle upon Tyne, UK); R. Williams (Freeman Hospital, Newcastle upon Tyne, UK); L. Wilson (Freeman Hospital, Newcastle upon Tyne, UK); M. G. Wyatt (Freeman Hospital, Newcastle upon Tyne, UK); W. Al-Jundi (Norfolk and Norwich University Hospital, Norwich, UK); I. Aziz (Norfolk and Norwich University Hospital, Norwich, UK); C. Knight (Norfolk and Norwich University Hospital, Norwich, UK); N. Mohammed (Norfolk and Norwich University Hospital, Norwich, UK); P. Stather (Norfolk and Norwich University Hospital, Norwich, UK); O. Agu (Royal Free Hospital, London, UK); C. Bishop (University College London Hospital, London, UK); D. Boardley (University College London Hospital, London, UK); J. Constantinou (Royal Free Hospital, London, UK); J. Cross (University College London Hospital, London, UK); M. Davis (Royal Free Hospital, London, UK); C. Eng (Royal Free Hospital, London, UK); R. Gumama (Royal Free Hospital, London, UK); J. Hague (University College London Hospital, London, UK); G. Hamilton (Royal Free Hospital, London, UK); P. L. Harris (University College London Hospital, London, UK); K. Ivancev (University College London Hospital, London, UK); J. Raja (University College London Hospital, London, UK); T. Richards (University College London Hospital, London, UK); D. Simring (University College London Hospital, London, UK); Y. Uddin (Royal Free Hospital, London, UK); N. Elzefzaf (Southampton General Hospital, Southampton, UK); G. Gamtkitsulashvili (Southampton General Hospital, Southampton, UK); S. Mathew (Southampton General Hospital, Southampton, UK); B. Patterson (Southampton General Hospital, Southampton, UK); C. Atkinson (St George’s Hospital, London, UK); B. Azhar (St George’s Hospital, London, UK); J. Budge (St George’s Hospital, London, UK); R. Furlong (St George’s Hospital, London, UK); I. Loftus (St George’s Hospital, London, UK); T. Loosemore (St George’s Hospital, London, UK); R. Morgan (St George’s Hospital, London, UK); A. Pouncey (St George’s Hospital, London, UK); I. Roy (St George’s Hospital, London, UK); M. M. Thompson (St George’s Hospital, London, UK); C. Bicknell (Imperial College Healthcare NHS Trust, London, UK); P. Bourke (Imperial College Healthcare NHS Trust, London, UK); N. Cheshire (Imperial College Healthcare NHS Trust, London, UK); I. Franklin (Imperial College Healthcare NHS Trust, London, UK); R. Gibbs (Imperial College Healthcare NHS Trust, London, UK); M. Hamady (Imperial College Healthcare NHS Trust, London, UK); A. James (Imperial College Healthcare NHS Trust, London, UK); M. P. Jenkins (Imperial College Healthcare NHS Trust, London, UK); C. Riga (Imperial College Healthcare NHS Trust, London, UK); S. Salim (Imperial College Healthcare NHS Trust, London, UK); M. Abdelhalim (Guy’s and St Thomas’ Hospital, London, UK); S. Abisi (Guy’s and St Thomas’ Hospital, London, UK); S. Black (Guy’s and St Thomas’ Hospital, London, UK); T. W. Carrell (Guy’s and St Thomas’ Hospital, London, UK); M. Dialynas (Guy’s and St Thomas’ Hospital, London, UK); P. Gkoutzios (Guy’s and St Thomas’ Hospital, London, UK); B. Modarai (Guy’s and St Thomas’ Hospital, London, UK); T. Sabharwal (Guy’s and St Thomas’ Hospital, London, UK); R. Salter (Guy’s and St Thomas’ Hospital, London, UK); R. Sandford (Guy’s and St Thomas’ Hospital, London, UK); M. R. Tyrrell (King’s College Hospital, London, UK); M. Waltham (Guy’s and St Thomas’ Hospital, London, UK); C. J. Wilkins (King’s College Hospital, London, UK).
