# Supplementary material for: Nationwide outcomes of fenestrated endovascular aneurysm repair
Source: Br J Surg. 2026 Mar 27;113(5):znag037. doi: 10.1093/bjs/znag037 (PMC13167452; doi:10.1093/bjs/znag037)
Supplement: znag037_Supplementary_Data [file znag037_supplementary_data.zip › Lay_summary.docx]

**This lay summary has been written with PPIE expert input**

**Background- why are we doing this research?**

An Abdominal Aortic Aneurysm (AAA) is a swelling of the main blood vessel in the abdomen (tummy) and affects thousands of people each year. AAAs can grow over time and may burst.

If the swelling gets too large, people are offered an operation to repair their swollen blood vessel to stop it bursting. This used to involve an operation that cut open your abdomen but now surgeons can use keyhole methods to place a small metal mesh tube (stent) inside the swollen blood vessel instead.

AAAs come in all shapes and sizes, and around a third of AAAs have awkward shapes that need a ‘complex’ keyhole method called a Fenestrated Endovascular Aneurysm Repair (FEVAR). In this operation, the stent needs to be custom-made to fit the awkward shape of the AAA, rather than using an off the shelf stent which can be used in more common AAA shapes.

Despite thousands of complex repairs taking place each year world-wide, we do not know what the long-term recovery after this operation is or if people live as long as people having other types of operations.

To try to understand this, 15 UK hospitals have teamed up to record when they do these complex operations, how patients recover afterwards and if they need further operations. This register is called GLOBALSTAR.

**Aims**

1. Finding out the long-term results for FEVAR:

a. How long do people live for?

b. How many people have a re-operation?

2. How do older patients over 80 years-old fare after the operation?

3. How do women fare after the operation?

**Methods**

We used information in the registry and statistics to describe the long-term results for FEVAR.

**Findings and conclusion- what did we find?**

We found that after the operation, people will live on average 7 years, and around a quarter will need a re-operation within 7 years. The main reason for needing a re-operation are technical issues with the device itself, which accounts for three quarters of reasons to re-operate. The procedure was riskier for women, with twice the risk of a complication immediately after the operation compared to men. In contrast, eighty-year-olds had a similar level of risk related to surgery compared to younger patients and around 90% were still alive at 1 year after the operation. Eighty-year-olds survived over 5 years on average, this probably means that patients and doctors are good at deciding who should go forward with surgery in this age group. This study suggests that FEVAR is a good option for repairing AAAs in the right people. Lowering the risk of re-operation is really important: planning the operation carefully and checking up on patients to pick up any issues with the device are important areas to improve on. Getting these things right will make care for people with AAAs better.
