# Supplementary material for: Nationwide outcomes of fenestrated endovascular aneurysm repair
Source: Br J Surg. 2026 Mar 27;113(5):znag037. doi: 10.1093/bjs/znag037 (PMC13167452; doi:10.1093/bjs/znag037)
Supplement: znag037_Supplementary_Data [file znag037_supplementary_data.zip › Supplementary_Material.docx]

**Title**

**Nationwide outcomes of Fenestrated Endovascular Aneurysm Repair**

Authors:

Aurélien M Guéroult^1, 2^ on behalf of the British Society of Endovascular Surgery and the GLOBALSTAR Collaborators

1. St George’s Vascular Institute, City St George’s University of London
2. School of Health and Medical Sciences, City St George’s University of London

***GLOBALSTAR Collaborators:***

*A. M. Guéroult^++^, P. Holt, M. Juszczak, S. Neequaye^+^.*

*S. Dindyal, V. Gadhvi, M. Hossain^§^, A. Kordzadeh. D. Adam, M. Claridge, M. Hook, K. Powezka, M. Vezzosi. P. Bevis, M. Brooks, M. Dewi, J. Hardman. G. Ambler, A. Awopetu^§^, J.R. Boyle, C. Cousins, P.D. Hayes, T. Mehta, G. Penney^§^, T.C. See, K. Varty, A. Winterbottom. M. Bown, E. Choke, M. McCarthy, A. Saratzis^§^, R. Sayers, A. Tambyraja. J .A. Brennan, R. Canavati, R.K. Fisher, S. Holder^§^, R.G. McWilliams, J.B. Naik, S. Vallabhaneni^*^. A. Alshiekh^§^, F. Farquharson, F. Serracino-Inglott. R. Bell, A. Burdess, M.J. Clarke, T. El-Sayed^§^, R. Jackson, J. McCaslin, S. Nandhra, J.D. Rose, A. Sharif, V. Wealleans, R. Williams, L. Wilson, M.G. Wyatt. W. Al-Jundi, I. Aziz^§^, C. Knight, N. Mohammed, P. Stather. O. Agu, C. Bishop, D. Boardley, J. Constantinou, J. Cross, M. Davis, C. Eng^§^, R. Gumama, J. Hague, G. Hamilton, P.L. Harris, K. Ivancev, J. Raja, T. Richards, D. Simring, Y. Uddin. N. Elzefzaf, G. Gamtkitsulashvili^§^, S. Mathew, B. Patterson. C. Atkinson^§^, B. Azhar, S. Black, J. Budge, R. Furlong^§^, R. Hinchliffe, I. Loftus, T. Loosemore, R. Morgan, A. Pouncey, I. Roy, M.M. Thompson. C. Bicknell, P. Bourke, N. Cheshire, I. Franklin, R. Gibbs, M. Hamady, A. James, M.P. Jenkins, C. Riga, S. Salim^§^. M. Abdelhalim, S. Abisi, T.W. Carrell, M. Dialynas, P. Gkoutzios, B. Modarai^$^, T. Sabharwal, R. Salter, R. Sandford, M.R. Tyrrell, M. Waltham, C.J. Wilkins. S. Hobbs.*

^+^Chief Investigator, ^++^Co-investigator, ^*^Registry founder, ^$^President of BSET, ^§^Significant contribution to data collection.

**Corresponding author.** Name and address **ORCID ID**; **Twitter**

Aurélien M Guéroult

BA (Path), MB BChir, MA (Cantab), MMedEd, PGCert HRSM, MRCS (Eng)

[Aurelien.gueroult1@nhs.net](mailto:Aurelien.gueroult1@nhs.net)

St George’s Vascular Institute, City St George's, University of London, Tooting campus, London SW17 0RE.

ORCID: 0000-0002-5042-0455

**Supplementary Materials - Index**

| **Supplementary Methods** |  |
| --- | --- |
| Methods for sensitivity analyses | *page 3* |
| Statistical methods and code | *pages 3-11* |
| **Supplementary Results** |  |
| Results of sensitivity analyses | *page 12* |
| **Supplementary Appendixes** |  |
| **Appendix 1.** Fields in the GLOBALSTAR data collection protocol. | *pages 13-15* |
| **Appendix 2.** GLOBALSTAR collaborators list. | *page 16* |
| **Supplementary Figures and Tables** |  |
| **Supplementary figure 1.** GLOBALSTAR study flow diagram. | *page 17* |
| **Supplementary figure 2.** Cumulative incidence of endoleak types with all-cause mortality as a competing risk. | *page 18* |
| **Supplementary figure 3.** Cumulative incidence of loss of target vessel patency with all-cause mortality as a competing risk. | *page 19* |
| **Supplementary figure 4.** Subgroup analysis of survival by sex. | *Page 20* |
| **Supplementary figure 5.** Subgroup analysis of survival by age-group (octogenarians versus non-octogenarians). | *Page 21* |
| **Supplementary figure 6.** Forest plot for multivariable logistic regression sensivity analysis: 1-year all-cause mortality. | *Page 22* |
| **Supplementary figure 7.** Forest plot for multivariable logistic regression sensivity analysis: 5-year all-cause mortality. | *Page 23* |
| **Supplementary figure 8.** Chronologically-stratified Kaplan Meier analysis for survival. | *Page 24* |
| **Supplementary figure 9.** Chronologically-stratified cumulative incidence analysis for type 2 endoleaks with all-cause mortality as a competing risk. | *Page 25* |
| **Supplementary Table 1.** Contributions by collaborating centre. | *Page 26* |
| **References** | *page 27* |

**Supplementary Methods**

## Sensitivity analyses

Multivariable regression against survival at 1 and 5 years was performed as a sensitivity analysis. Variables included case recency, age at operation, sex, co-morbidities, smoking status, prior aortic surgery, ASA, pre-operative blood results, aneurysm diameter and centre contribution (binarised >50 cases contributed).

Collinearity between variables was assessed by calculating the Pearson Correlation coefficient and manually excluding the least clinically informative predictor in highly correlated pairs. Feature selection for multivariable modelling was performed by applying Cox univariable regression across imputed datasets for each candidate variable, assessing association to all-cause mortality at 1 and 5 years.^20,21^

## Statistical methods and code

All statistical analyses were performed in R statistical software.^12^

### Time-to-event analyses

R packages *survival,^21^ survminer^22^* and *ggplot2^23^* were used to perform Kaplan-Meier time-to-event analyses. The generic statistical code is presented below:

km_plot <- ggsurvplot(

survfit(Surv(Time, Death) ~ 1, data=data), # Kaplan-Meier survival curve

data = data,

pval = FALSE, # Include p-value

conf.int = TRUE, # Include confidence intervals

risk.table = FALSE, # Include a risk table

risk.table.title = "No. at Risk",

surv.median.line = "hv",

xlim = c(0, 10),

ggtheme = theme_minimal(),

break.time.by = 1,

xlab = "Time (years)",

ylab = "Survival",

legend.title = "Survival"

)

*Cmprsk^13^* package was used to perform cumulative incidence with competing risks for all-cause mortality. The generic statistical code is presented below:

# Cumulative incidence object

cuminc_resultre <- cmprsk::cuminc(ftime = Time, fstatus = Reint_CI)

# CI Plot for re-intervention

reintplot <- ggcompetingrisks(

fit = cuminc_resultre,

curvetype = "cuminc",

palette = c("red", "grey"),

xlab = "Time (years)",

ylab = "Cumulative incidence of Re-intervention",

legend.title = "Re-intervention",

legend.labs = "Re-intervention",

risk.table = TRUE,

xlim = c(0, 10),

ylim = c(0, 0.4),

conf.int = TRUE,

break.time.by = 1, # Yearly breaks on x-axis

ggtheme = theme_bw() # Clean theme

)

### Handling of missing data

Pre-operative variables with >5% missingness (table 1) were treated with multiple imputation by chained equations using the *mice* package.^20^

#PREPARE DATA

# All pre-operative variables

covariates <- c("recency", "Age_op", "sex", "Diabetes", "IHD", "CHF", "HTN", "CKD", "PAD", "Prior_surgery", "ASA", "Haemaglobin", "Sodium", "Potassium", "Urea", "Creatinine", "Aneurysm_diameter", "Smoker", "Centre_con")

#IMPUTATION

# Combine covariates and outcomes

all_vars <- c("Time", "Death", covariates)

impute_data <- df[, all_vars]

# Create method vector to impute only variables >5% missingness

meth <- make.method(impute_data)

meth["Time"] <- ""

meth["Death"] <- ""

meth["recency"] <- ""

meth["Age_op"] <- ""

meth["sex"] <- ""

meth["Diabetes"] <- ""

meth["IHD"] <- ""

meth["CHF"] <- ""

meth["HTN"] <- ""

meth["CKD"] <- ""

meth["Aneurysm_diameter"] <- ""

meth["Centre_con"] <- ""

# Run imputation, excluding Time and Death from being imputed

imputed_data <- mice(impute_data, m = 5, method = meth, seed = 500)

# Extract imputed datasets (list of data frames)

completed_datasets <- lapply(1:imputed_data$m, function(i) complete(imputed_data, i))

### Sensitivity analyses

Multivariable regression against survival at 1 and 5 years was performed as a sensitivity analysis. Feature selection for multivariable modelling was performed by applying Cox univariable regression across imputed datasets for each candidate variable, assessing association to all-cause mortality at 1 and 5 years.^20,21^

# Create a list to store results for each time point

pooled_results <- list()

# Loop through each covariate and perform univariate Cox regression for survival at 1 and 5 years

for (var in covariates) {

# For each covariate, fit the univariate Cox model for each imputed dataset at 1 and 5 years

for (t in 1,5) { # Loop over time points (1-5 years)

# Fit the Cox model for the specific covariate across imputed datasets

models <- lapply(completed_datasets, function(data) {

# Create a new column to represent whether the individual survived beyond time t

# Right-censor data at time t: If Time > t, censor at t

data$Time_at_t <- pmin(data$Time, t)

data$Death_at_t <- ifelse(data$Time <= t & data$Death == 1, 1, 0) # Only count death events within time t

# Adjust the survival formula to account for survival at specific time point

formula <- as.formula(paste0("Surv(Time_at_t, Death_at_t) ~ ", var))

# Fit the Cox model for the specific covariate

cox_model <- coxph(formula, data = data)

return(cox_model)

})

# Convert models to 'mira' class for pooling

mira_obj <- as.mira(models)

# Pool results across imputed datasets

pooled <- pool(mira_obj)

# Extract summary statistics

summ <- summary(pooled, conf.int = TRUE)

# Store results for this time point

pooled_results[[paste0(var, "_", t, "years")]] <- data.frame(

Variable = var,

Time_Point = paste0(t, " years"),

beta = round(summ$estimate, 2),

HR = round(exp(summ$estimate), 2), # Exponentiated coefficient for HR

CI_lower = round(exp(summ$`2.5 %`), 2), # Exponentiated lower CI

CI_upper = round(exp(summ$`97.5 %`), 2), # Exponentiated upper CI

p_value = round(summ$p.value, 3)

)

}

}

Candidate predictors were selected at 1 and 5 years based on a threshold p< 0.1.

# Step: Select significant predictors (p < 0.1) at 1 year

selected_vars_1yr <- univ_results_df[

univ_results_df$Time_Point == "1 years" & univ_results_df$p_value < 0.1,

"Variable"

]

# Remove duplicates in case same var was tested multiple times

selected_vars_1yr <- unique(as.character(selected_vars_1yr))

print(selected_vars_1yr)

A variety of models were applied using selected features, including cox multivariable, penalised likelihood, deep neural network, random forest, XG boost and logistic regression. None of these models were found to perform particularly well; multivariable logistic regression was found to be the most performant in terms of discrimination and calibration. Therefore, logistic regression (generalised linear model) was applied to perform sensitivity analyses across imputed datasets. Code for model assessment by discrimination and calibration is provided below.^24^

#DISCRIMINATION

logit_models <- list()

logit_predictions <- list()

logit_aucs <- numeric(length(completed_datasets))

for (i in seq_along(completed_datasets)) {

data_i <- completed_datasets[[i]]

# Binary target: died within 1 year

data_i$event_1yr <- as.numeric(data_i$Time <= 1 & data_i$Death == 1)

# Ensure selected predictors are available

usable_vars <- intersect(selected_vars_1yr, colnames(data_i))

if (length(usable_vars) == 0) {

warning(paste("No usable predictors in dataset", i))

next

}

# Drop rows with NA in selected vars or outcome

model_data <- data_i[, c(usable_vars, "event_1yr")]

model_data <- model_data[complete.cases(model_data), ]

if (nrow(model_data) < 10) {

warning(paste("Too few complete cases in dataset", i))

next

}

# Build logistic model formula

form <- as.formula(paste("event_1yr ~", paste(usable_vars, collapse = " + ")))

# Fit logistic regression

model <- glm(form, data = model_data, family = "binomial")

# Predict probabilities

preds <- predict(model, type = "response")

true <- model_data$event_1yr

# Store model and AUC

logit_models[[i]] <- model

logit_predictions[[i]] <- preds

logit_aucs[i] <- auc(roc(true, preds))

}

# Report mean AUC

mean_logit_auc <- mean(logit_aucs, na.rm = TRUE)

#CALIBRATION

# Create design object for rms

dd <- datadist(model_data)

options(datadist = 'dd')

# Fit logistic model using rms

formula_str <- paste("event_1yr ~", paste(usable_vars, collapse = " + "))

logit_rms <- lrm(as.formula(formula_str), data = model_data, x = TRUE, y = TRUE)

# Plot calibration curve

cal <- calibrate(logit_rms, method = "boot", B = 200)

plot(cal, xlab = "Predicted Probability", ylab = "Observed Probability",

main = "Calibration Curve - Logistic Model")

Odd ratio tables and associated forest plots (pooled across imputations) were generated for logistic regression models.

logit_model_summaries <- lapply(logit_models, tidy, conf.int = TRUE, exponentiate = TRUE)

# Add model index

for (i in seq_along(logit_model_summaries)) {

logit_model_summaries[[i]]$model_id <- i

}

# Combine into one data frame

logit_all_models <- do.call(rbind, logit_model_summaries)

# Calculate pooled statistics: mean ORs and confidence intervals across models

logit_summary_pooled <- logit_all_models %>%

dplyr::group_by(term) %>%

dplyr::summarise(

OR = round(mean(estimate, na.rm = TRUE), 2),

CI_lower = round(mean(conf.low, na.rm = TRUE), 2),

CI_upper = round(mean(conf.high, na.rm = TRUE), 2),

p_value = round(mean(p.value, na.rm = TRUE), 3),

.groups = "drop"

) %>%

dplyr::mutate(`OR (95% CI)` = paste0(OR, " (", CI_lower, "-", CI_upper, ")")) %>%

dplyr::select(term, `OR (95% CI)`, p_value)

# View final table

print(logit_summary_pooled)

#Forest plot

ggplot(logit_summary_pooled, aes(x = OR, y = term)) +

geom_point(size = 2.5) +

geom_errorbarh(aes(xmin = CI_lower, xmax = CI_upper), height = 0.2) +

geom_vline(xintercept = 1, linetype = "dashed", color = "red") +

labs(

title = "Pooled Odds Ratios (per 10-unit increase)",

x = "Odds Ratio",

y = NULL

) +

theme_minimal(base_size = 13)

Chronologically stratified time-to-event analyses were also performed to specifically examine the effect of case recency on outcomes. Example code is provided below.

#SURVIVAL (KM)

data <- MORTALITY_MASTER %>%

mutate(

OperationDate = as.Date(`Operation Date`),

CaseYear = year(OperationDate),

era = cut(

CaseYear,

breaks = c(2002, 2008, 2013, 2019, Inf), # 2003–08, 09–14, 15–20, 2021+

labels = c("2003–2008", "2009–2013", "2014–2019", "2020+"),

right = TRUE, include.lowest = TRUE

)

) %>%

filter(!is.na(era))

# Make sure era has clean levels (trim spaces, normalize dashes if needed)

levels(data$era) <- trimws(levels(data$era))

# Build an UNNAMED palette with one color per era level (order matters)

era_lvls <- levels(data$era)

pal <- c("#E41A1C", "#377EB8", "#4DAF4A", "#984EA3")[seq_along(era_lvls)]

fit_era <- survfit(Surv(Time, Death) ~ era, data = data)

km_era <- ggsurvplot(

fit_era,

data = data,

conf.int = TRUE,

risk.table = TRUE,

risk.table.title = "No. at risk",

break.time.by = 1,

xlim = c(0, 4),

ggtheme = theme_minimal(),

pval= TRUE,

pval.method = TRUE,

xlab = "Time (years)",

ylab = "Survival",

legend.title = "Case recency",

palette = pal # <- unnamed vector; no more mismatch warning

)

print(km_era)

# Censor follow-up at 4y

data_4y <- data %>%

mutate(

Time4 = pmin(Time, cutoff),

Death4 = ifelse(Time <= cutoff & Death == 1, 1, 0) # deaths after 4y become censored at 4

)

# Log-rank test across eras at 4y

lr5 <- survdiff(Surv(Time4, Death4) ~ era, data = data_4y)

p_lr4 <- 1 - pchisq(lr4$chisq, df = nlevels(data_4y$era) - 1)

cat(sprintf("Log-rank at %d years across eras: Chi^2 = %.3f, df = %d, p = %.4f\n",

cutoff, lr4$chisq, nlevels(data_4y$era) - 1, p_lr4))

pw <- pairwise_survdiff(Surv(Time4, Death4) ~ era, data = data_4y, p.adjust.method = "none")

print(pw$p.value) # unadjusted pairwise p-values at 4y

#CUMULATIVE INCIDENCE

# --- Build CaseYear and era from `Operation Date`

data2 <- REINT_CI_FINAL %>%

mutate(

OperationDate = as.Date(`Operation Date`), # safe even if already Date

CaseYear = year(OperationDate),

era = cut(

CaseYear,

breaks = c(2002, 2008, 2013, 2019, Inf), # 2003–08, 09–14, 15–20, 2021+

labels = c("2003–2008", "2009–2013", "2014–2019", "2020+"),

right = TRUE,

include.lowest = TRUE

)

) %>%

filter(!is.na(era))

cuminc_resultre <- cmprsk::cuminc(

ftime = data2$Time,

fstatus = data2$Reint_CI,

group = data2$era,

cencode = 0

)

# fit CIF with competing risks, stratified by era

# Assumes: data2$Time is in years; Reint_CI: 0=censored, 1=re-intervention, others=competing

cuminc_resultre <- cmprsk::cuminc(

ftime = data2$Time,

fstatus = data2$Reint_CI,

group = data2$era,

cencode = 0

)

# plot event 1 (re-intervention) by era with 95% CI up to 4y

tidy_cuminc <- function(ci_obj) {

keep <- setdiff(names(ci_obj), c("Tests", "n"))

out <- lapply(keep, function(nm) {

x <- ci_obj[[nm]]

df <- data.frame(time = x$time, est = x$est, var = x$var, curve = nm)

df

})

d <- dplyr::bind_rows(out)

d <- d %>%

mutate(

event_front = suppressWarnings(as.integer(sub("^([0-9]+)\\s+.*$", "\\1", curve))),

event_back = suppressWarnings(as.integer(sub("^.*\\s+([0-9]+)$", "\\1", curve))),

event = ifelse(!is.na(event_front), event_front, event_back),

group = ifelse(!is.na(event_front),

sub("^[0-9]+\\s+", "", curve),

sub("\\s+[0-9]+$", "", curve))

)

d

}

dcurves <- tidy_cuminc(cuminc_resultre) %>%

dplyr::filter(event == 1) %>%

mutate(

lower = pmax(0, est - qnorm(0.975) * sqrt(var)),

upper = pmin(1, est + qnorm(0.975) * sqrt(var))

)

p <- ggplot(dcurves %>% dplyr::filter(time <= 10),

aes(x = time, y = est, colour = group, fill = group)) +

geom_step() +

geom_ribbon(aes(ymin = lower, ymax = upper), alpha = 0.12, colour = NA) +

scale_x_continuous("Time (years)", breaks = 0:4, limits = c(0, 4)) +

scale_y_continuous("Cumulative incidence of Re-intervention", limits = c(0, 0.4)) +

labs(colour = "Case recency", fill = "Case recency",

title = "Cumulative incidence of re-intervention by case recency") +

theme_bw()

print(p)

#Gray’s test at 4 years across time periods

ci_5y <- cmprsk::cuminc(

ftime = data2_5y$Time4,

fstatus = data2_5y$Reint_CI_4,

group = data2_5y$era,

cencode = 0

)

tests_4y <- ci_4y$Tests

row1 <- which(rownames(tests_4y) == "1")

cat("\nGray's test at 4 years for event 1 across eras:\n")

if (length(row1)) {

print(tests_4y[row1, , drop = FALSE])

} else {

cat("No event-1 test row found (possibly no event-1 before 4y in at least one era).\n")

}

**Supplementary Results**

### Sensitivity analyses results

#### 1-year mortality

Results of logistic regression time-stamped at 1 year are presented in supplementary figure 6. These results show a statistically significant association between 1-year mortality and female sex OR= 2.01 (95% CI, 1.16-3.35, p= 0.009) and age at operation was not statistically significantly associated with increased odds of mortality.

Pooled discrimination assessed by Area Under the Curve (AUC)= 0.66 (fair) and calibration data for the first imputed dataset: n=1522, Mean absolute error=0.007, Quantile of absolute error=0.011.

#### 5-year mortality

Results of logistic regression time-stamped at 5 years are presented in supplementary figure 7. These results show a statistically significant association between haemoglobin and a lower odds of mortality at 5 years; and between prior aortic surgery, creatinine, age, aneurysm diameter and an increased odds of mortality at 5 years.

Pooled discrimination assessed by Area Under the Curve (AUC)= 0.63 (fair) and calibration data for the first imputed dataset: n=1522, Mean absolute error=0.005, Quantile of absolute error=0.015.

**Supplementary Appendixes**

## **Appendix 1.** Fields in the GLOBALSTAR data collection protocol.

Case Registration Data

Centre Identifier Name of Centre case performed In (Auto filled)
Registered By User ID Registering Case (Auto filled)
Physician Identifier Physician Undertaking Procedure
Patient Date of Birth Date (Available to own care team only)
Operation Date Date
Operation Type Pre-selected list of Complex Aneurysm repairs

Pre-operative (Patient) Data

Gender Male/Female (1/2)
Height Height in cm
Weight Weight in Kg
Smoking Status Smoker, Ex-Smoker, Non-Smoker (1,2,0)
Known Ischemic Heart Disease Yes/No (1/0)
Known Diabetes Yes/No (1/0)
Known Hypertension Yes/No (1/0)
Known Congestive Cardiac Failure Yes/No (1/0)
Known Chronic Renal Impairment Yes/No (1/0)
Known Cerebrovascular Disease Yes/No (1/0)
Known Prior aortic surgery Yes/No (1/0)
ASA Grade Numeric Value
Pre-operative Systolic Blood Pressure Numeric Value
Pre-operative Pulse Rate Numeric Value
Pre-operative Haemoglobin Numeric Value
Pre-operative Sodium Numeric Value
Pre-operative Potassium Numeric Value
Pre-operative Urea Numeric Value
Pre-operative Creatinine Numeric Value
Pre-operative ECG AF / Other Abnormality / Normal
FEV1 Numeric Value
Left Ejection Fraction Numeric Value
Aneurysm Size (mm) Numeric Value

Operative (Stent-graft) Data

Target Vessel Modality & Stenting:
 Celiac Axis Scallop/Fenestration & Stented/Un-Stented
 Superior Mesenteric Artery Scallop/Fenestration & Stented/Un-Stented
 Left Renal Artery Scallop/Fenestration & Stented/Un-Stented
 Right Renal Artery Scallop/Fenestration & Stented/Un-Stented
Device & Stent Design Free text Box
Operation Time Numeric Value
Total Blood Loss Numeric Value

Intra-operative Adjunctive Manoeuvres Yes/No
Describe Manoeuvres Free Text Box
Endoleak on Completion:
 Type I Proximal Yes/No
 Type I Distal Yes/No
 Type II Yes/No
 Type III TV stent connection Yes/No
 Type III Bodies Connection Yes/No
 Type III Limb Connections Yes/No
Any Unplanned Graft Use Yes/No
Describe Unplanned Graft Use Free Text Box
Target Vessels Fully Patent on Completion Yes/No
Describe Target Vessel loss Free Text Box

Post-Operative Data

ICU Days Numeric Value
Hospital Stay Numeric Value
Discharge Date Date
Needed Dialysis Yes/No
Any Post-op Morbidity Yes/No
Describe post-op morbidity Free Text Box
Date of Discharge Date
Date of Death Date
Secondary Intervention Before Discharge Yes/No
Indication Free Text Box
Nature of Intervention Free Text Box
Outcome Free Text Box
Complications Yes/No
Complication description Free Text Box
Patient Died Yes/No

Follow-up Data – This data can be recorded multiple times per / patient

Follow-up Date Date
Duplex Date Date
CT Date Date
X-ray Date Date
Target Vessels Status Satisfactory/Threatened /Occluded
Target Vessel Details Free Text Box
Aneurysm Diameter Numeric Value
Complications:
 Migration Yes/No
 Stent-graft Distortion Yes/No
 Limb Occlusion Yes/No
 Target Vessel Stent Distortion Yes/No
 Endoleak Type I Yes/No
 Endoleak Type II Yes/No
 Endoleak Type III Yes/No
 Endoleak Undetermined Yes/No
 Modular Distraction Yes/No
 Intra-Branch Distraction Yes/No
Episode Number Numerical
Creatinine Checked Date
Creatinine Value
Secondary Intervention Performed Yes/No
Secondary Intervention Description Free Text Box

**Appendix 2.** GLOBALSTAR collaborators list.

**GLOBALSTAR Collaborators:**

*GLOBALSTAR study steering group:* A. M. Guéroult^++^, P. Holt, M. Juszczak, S. Neequaye^+^*.*

*Basildon:* S. Dindyal, V. Gadhvi, M. Hossain^§^, A. Kordzadeh. *Birmingham:* D. Adam, M. Claridge, M. Hook, K. Powezka, M. Vezzosi. *Bristol:* P. Bevis, M. Brooks, M. Dewi, J. Hardman. *Cambridge:* G. Ambler, A. Awopetu^§^, J.R. Boyle, C. Cousins, P.D. Hayes, T. Mehta, G. Penney^§^, T.C. See, K. Varty, A. Winterbottom. *Leicester:* M. Bown, E. Choke, M. McCarthy, A. Saratzis^§^, R. Sayers, A. Tambyraja. *Liverpool:* J.A. Brennan, R. Canavati, R.K. Fisher, S. Holder^§^, R.G. McWilliams, J.B. Naik, S. Vallabhaneni^*^. *Manchester:* A. Alshiekh^§^, F. Farquharson, F. Serracino-Inglott. *Newcastle:* R. Bell, A. Burdess, M.J. Clarke, T. El-Sayed^§^, R. Jackson, J. McCaslin, S. Nandhra, J.D. Rose, A. Sharif, V. Wealleans, R. Williams, L. Wilson, M.G. Wyatt. *Norwich:* W. Al-Jundi, I. Aziz^§^, C. Knight, N. Mohammed, P. Stather. *Royal Free Hospital/ University College Hospital (London):* O. Agu, C. Bishop, D. Boardley, J. Constantinou, J. Cross, M. Davis, C. Eng^§^, R. Gumama, J. Hague, G. Hamilton, P.L. Harris, K. Ivancev, J. Raja, T. Richards, D. Simring, Y. Uddin. *Southampton:* N. Elzefzaf, G. Gamtkitsulashvili^§^, S. Mathew, B. Patterson. *St George’s (London):* C. Atkinson^§^, B. Azhar, S. Black, J. Budge, R. Furlong^§^, R. Hinchliffe, I. Loftus, T. Loosemore, R. Morgan, A. Pouncey, I. Roy, M.M. Thompson. *St Mary’s (London):* C. Bicknell, P. Bourke, N. Cheshire, I. Franklin, R. Gibbs, M. Hamady, A. James, M.P. Jenkins, C. Riga, S. Salim^§^. *St Thomas’/ King’s College (London):* M. Abdelhalim, S. Abisi, T.W. Carrell, M. Dialynas, P. Gkoutzios, B. Modarai^$^, T. Sabharwal, R. Salter, R. Sandford, M.R. Tyrrell, M. Waltham, C.J. Wilkins. *Wolverhampton:* S. Hobbs.

^+^Chief Investigator, ^++^Co-investigator, ^*^Registry founder, ^$^President of BSET, ^§^Significant contribution to data collection.

**Supplementary Figures and Tables**

**Supplementary figure 1.** GLOBALSTAR study flow diagram.


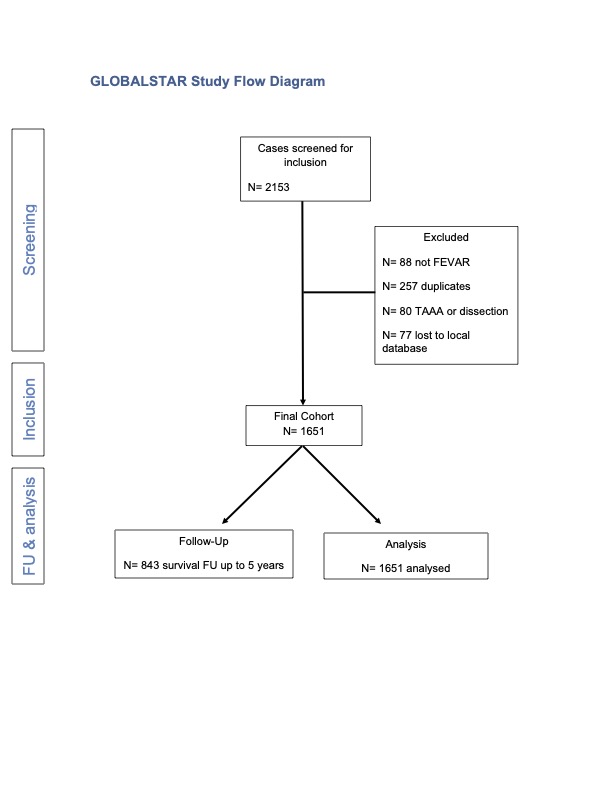


**Supplementary figure 2.** Cumulative incidence of endoleak types with all-cause mortality as a competing risk.


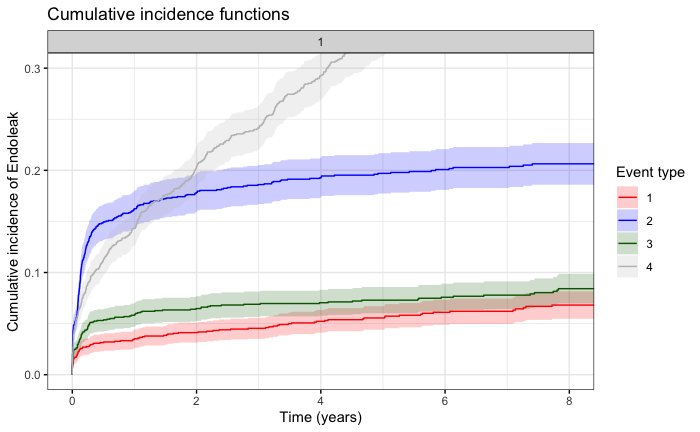


| **Time (years)** | **0** | **1** | **2** | **3** | **4** | **5** | **6** | **7** | **8** |
| --- | --- | --- | --- | --- | --- | --- | --- | --- | --- |
| N at risk | 1651 | 934 | 746 | 604 | 483 | 383 | 286 | 196 | 161 |
| Type 1a/1b endoleak (%) | 0 | 3.5 | 4.1 | 4.5 | 5.2 | 5.6 | 6.1 | 6.3 | 6.8 |
| 95% CI (%) | - | 2.6-4.4 | 3.2- 5.1 | 3.5- 5.6 | 4.1- 6.3 | 4.4- 6.7 | 4.9- 7.4 | 5.0- 7.5 | 5.4- 8.1 |
| Type 2 endoleak (%) | 0 | 16.2 | 17.8 | 18.6 | 19.3 | 19.7 | 20.1 | 20.3 | 20.6 |
| 95% CI (%) | - | 14.4- 18.0 | 15.9- 19.7 | 16.7- 20.5 | 17.4- 21.2 | 17.7- 21.7 | 18.1- 22.1 | 18.3- 22.3 | 18.6- 22.7 |
| Type 3 endoleak (%) | 0 | 5.8 | 6.4 | 6.9 | 7.0 | 7.3 | 7.6 | 7.8 | 8.4 |
| 95% CI (%) | - | 4.7- 7.0 | 5.2- 7.6 | 5.7- 8.3 | 5.8- 8.3 | 6.0- 8.6 | 6.2- 8.9 | 6.4- 9.1 | 6.9- 9.8 |

*Type 1a/1b endoleak in red, Type 2 endoleak in blue, Type 3 endoleak in green, all-cause mortality as a competing risk in grey, 95% CI ribbons. Shading in the number at risk table denotes end of data maturity.*

**Supplementary figure 3.** Cumulative incidence of loss of target vessel patency with all-cause mortality as a competing risk.


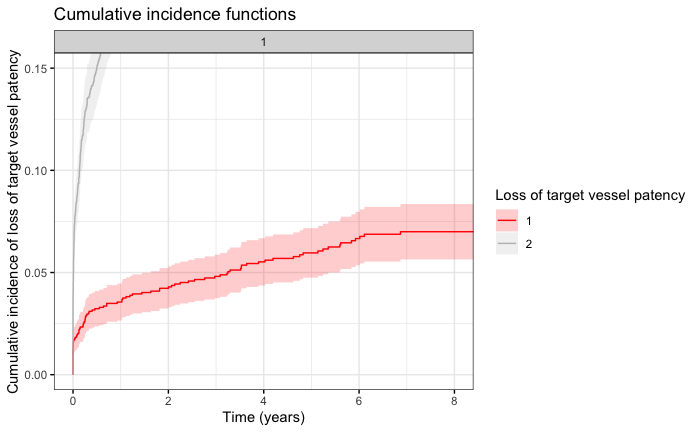


| **Time (years)** | **0** | **1** | **2** | **3** | **4** | **5** | **6** | **7** | **8** |
| --- | --- | --- | --- | --- | --- | --- | --- | --- | --- |
| N at risk | 1651 | 1175 | 999 | 829 | 668 | 499 | 393 | 287 | 287 |
| Loss of TVP(%) | 0 | 3.6 | 4.3 | 4.8 | 5.5 | 6.0 | 6.7 | 6.9 | 7.0 |
| 95% CI (%) | - | 2.7- 4.5 | 3.3- 5.3 | 3.7- 5.9 | 4.4- 6.7 | 4.8- 7.2 | 5.4- 8.0 | 5.6- 8.3 | 5.6- 8.4 |

*Loss of target vessel patency per individual in red, all-cause mortality as a competing risk in grey, 95% CI ribbons.*

**Supplementary figure 4.** Subgroup analysis of survival by sex.


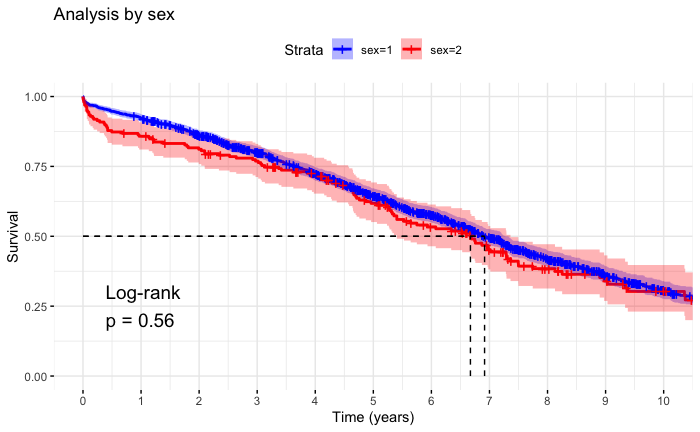


| **Time (years)** | **0** | **1** | **2** | **3** | **4** | **5** | **6** | **7** | **8** | **9** | **10** |
| --- | --- | --- | --- | --- | --- | --- | --- | --- | --- | --- | --- |
| N at risk (male) | 1433 | 1321 | 1197 | 1054 | 918 | 741 | 597 | 455 | 329 | 231 | 160 |
| Survival (%) | 100 | 92.3 | 86.1 | 80.0 | 72.2 | 64.5 | 57.7 | 49.5 | 41.8 | 36.0 | 30.7 |
| 95% CI (%) | - | 90.1- 93.6 | 84.3- 87.9 | 78.0- 82.1 | 69.8- 74.6 | 61.9- 67.1 | 55.0- 60.5 | 46.7- 52.5 | 38.9- 44.8 | 33.1- 39.2 | 27.8- 33.9 |
| N at risk (female) | 197 | 168 | 156 | 144 | 125 | 101 | 78 | 58 | 40 | 29 | 22 |
| Survival (%) | 100 | 85.8 | 81.1 | 76.9 | 71.4 | 61.8 | 53.4 | 45.2 | 38.4 | 34.1 | 30.3 |
| 95% CI (%) | - | 81.0- 90.8 | 75.8- 86.8 | 71.2- 83.1 | 65.3- 78.1 | 55.1- 69.3 | 46.4- 61.4 | 38.2- 53.6 | 31.3- 47.2 | 26.9- 43.2 | 23.0- 39.7 |
| Log-rank (p-value) | - | 0.002 | 0.04 | 0.2 | >0.05 | >0.05 | >0.05 | >0.05 | >0.05 | >0.05 | >0.05 |

*Survival for males in blue and females in red with 95% CI ribbon. Median survivals in dashed lines.*

**Supplementary figure 5.** Subgroup analysis of survival by age-group (octogenarians versus non-octogenarians).


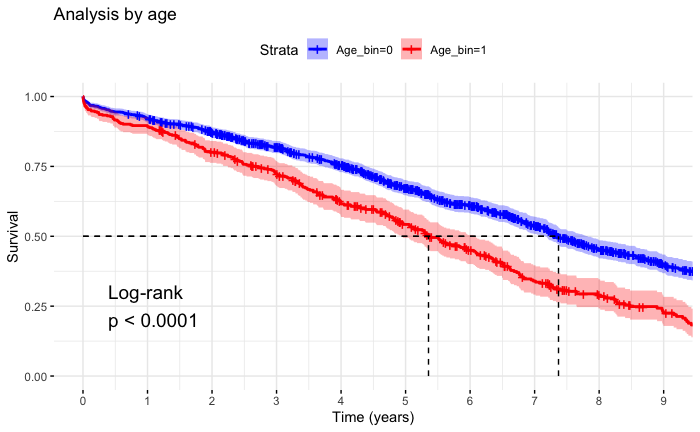


| **Time (years)** | **0** | **1** | **2** | **3** | **4** | **5** | **6** | **7** | **8** | **9** |
| --- | --- | --- | --- | --- | --- | --- | --- | --- | --- | --- |
| N at risk (<80 y.o.) | 1247 | 1146 | 1051 | 936 | 832 | 675 | 553 | 431 | 313 | 225 |
| Survival (%) | 100 | 92.1 | 87.1 | 81.9 | 75.4 | 67.4 | 61.1 | 53.8 | 45.2 | 40.0 |
| 95% CI (%) | - | 90.7- 93.6 | 85.2- 89.0 | 79.7- 84.1 | 73.0- 77.9 | 64.7- 70.1 | 58.3- 64.1 | 50.8- 57.0 | 42.1- 48.5 | 36.8- 43.4 |
| N at risk (≥80 y.o.) | 404 | 361 | 317 | 276 | 223 | 177 | 131 | 89 | 61 | 39 |
| Survival (%) | 100 | 89.4 | 80.1 | 72.4 | 61.6 | 54.2 | 45.0 | 34.0 | 28.9 | 22.5 |
| 95% CI (%) | - | 86.4- 92.4 | 76.3- 84.1 | 68.1- 76.9 | 56.9- 66.6 | 49.4- 59.5 | 40.1- 50.5 | 29.1- 39.6 | 24.2- 34.6 | 17.9- 28.3 |
| Log-rank (p-value) | - | 0.07 | <0.05 | <0.05 | <0.05 | <0.05 | <0.05 | <0.05 | <0.05 | <0.05 |

*Survival for non-octogenarians in blue and octogenarians in red with 95% CI ribbon. Median survivals in dashed lines. Shading in the number at risk table denotes end of data maturity.*

**Supplementary figure 6.** Forest plot for multivariable logistic regression sensivity analysis: 1-year all-cause mortality.


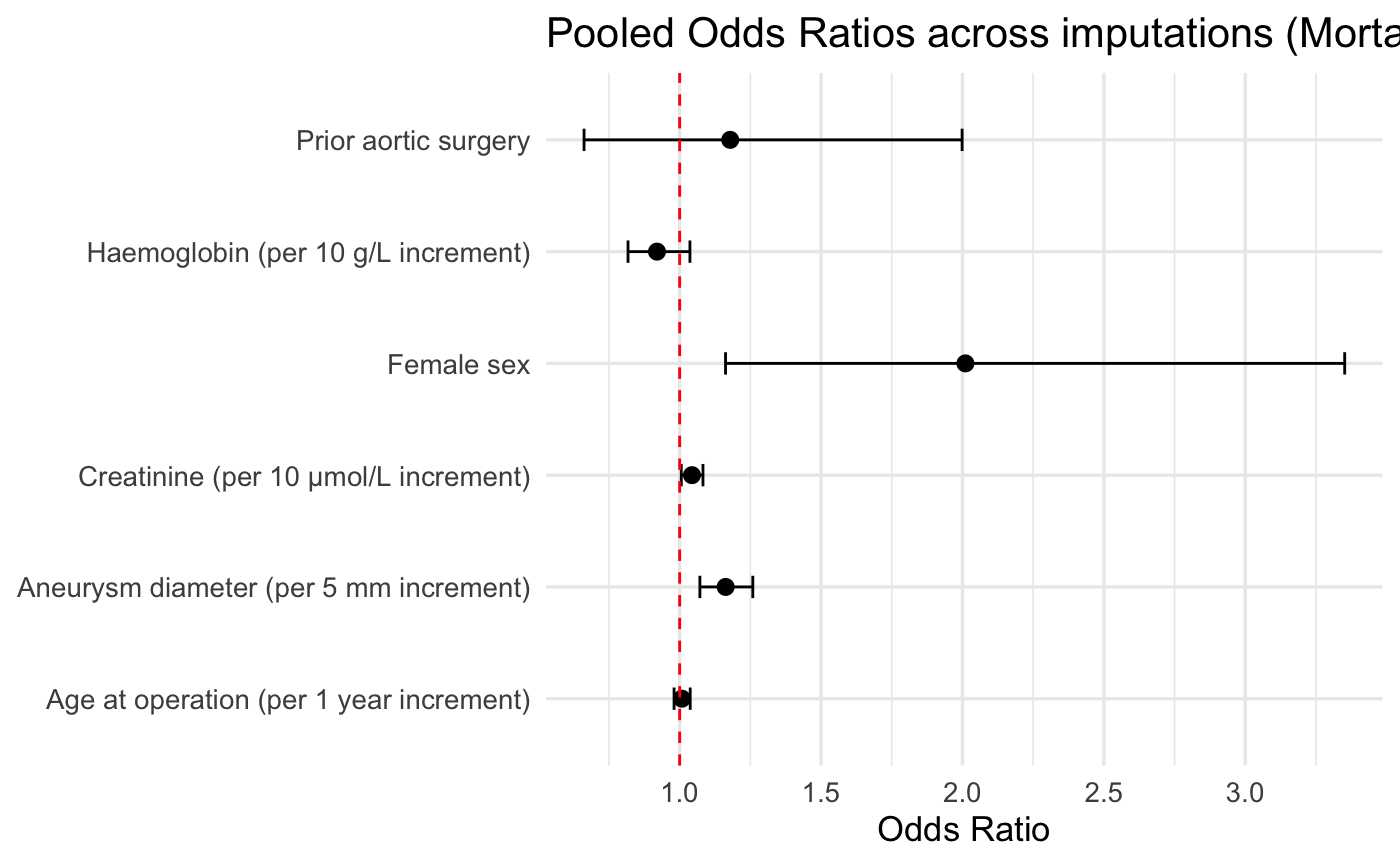


| Variables | OR (95% CI) | p value |
| --- | --- | --- |
| Prior aortic surgery | 1.18 (0.66-2) | 0.573 |
| Haemoglobin (per 10 g/L) | 0.92 (0.82-1.04) | 0.176 |
| Female sex | 2.01 (1.16-3.35) | 0.009 |
| Creatinine (per 10 umol/L) | - 1. (1.01-1.08) | 0.02 |
| Aneurysm diameter (per 5 mm) | 1.16 (1.07-1.26) | <0.001 |
| Age at operation (per year) | 1.01 (0.98-1.04) | 0.582 |

**Supplementary figure 7.** Forest plot for multivariable logistic regression sensivity analysis: 5-year all-cause mortality.


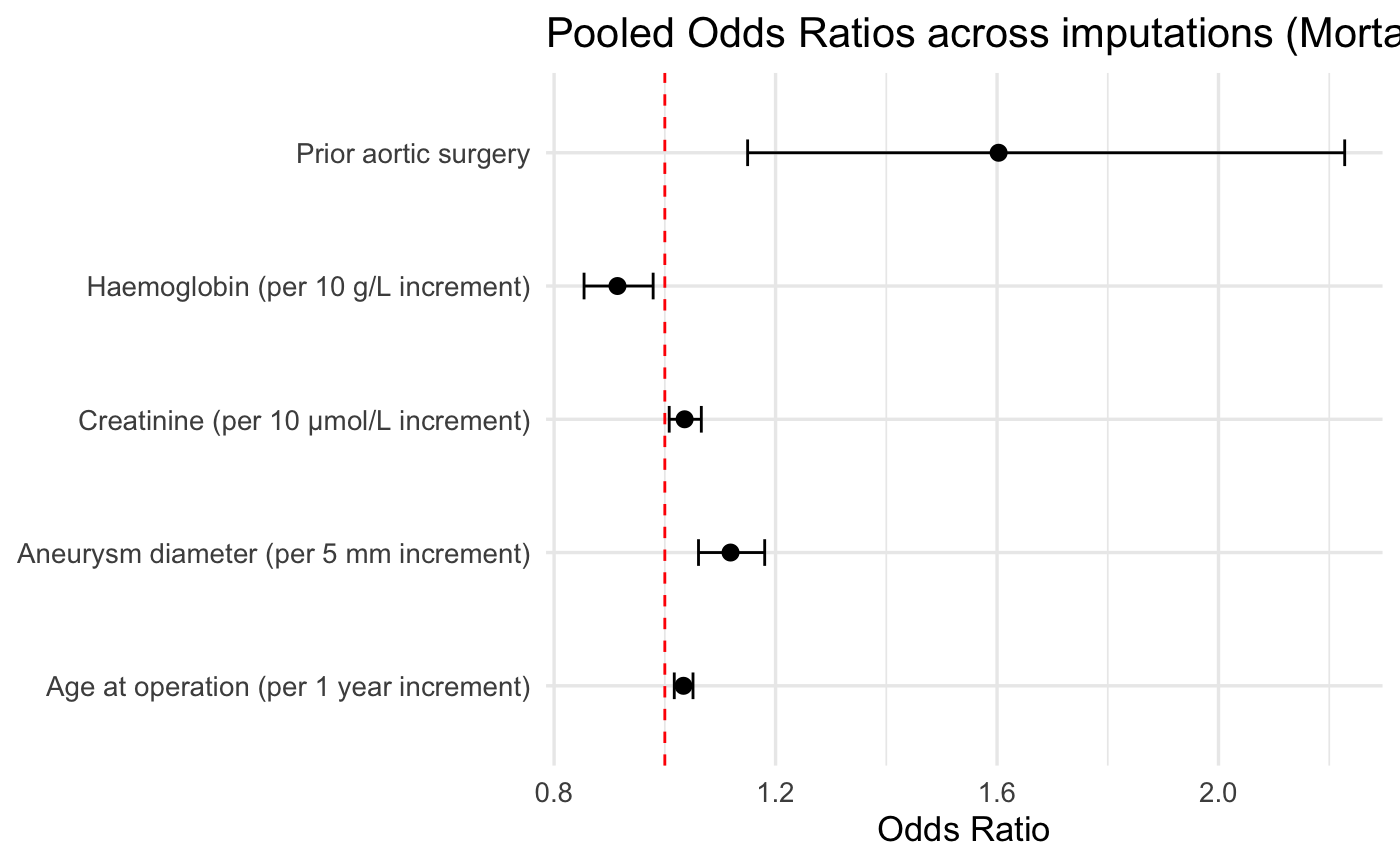


| Variables | OR (95% CI) | p value |
| --- | --- | --- |
| Prior aortic surgery | 1.6 (1.15-2.23) | <0.001 |
| Haemoglobin (per 10 g/L) | 0.91 (0.85-0.98) | 0.016 |
| Creatinine (per 10 umol/L) | 1.04 (1.01-1.07) | 0.016 |
| Aneurysm diameter (per 5 mm) | 1.12 (1.06-1.18) | <0.001 |
| Age at operation (per year) | 1.03 (1.02-1.05) | <0.001 |
|  |  |  |

**Supplementary figure 8.** Chronologically-stratified Kaplan Meier analysis for survival.


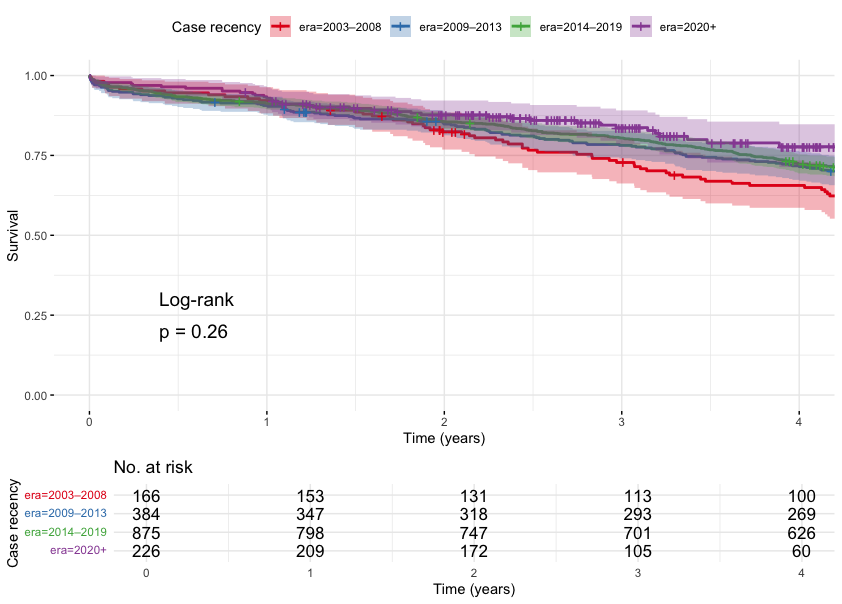


|  | 2003–2008 | 2009–2013 | 2014–2019 |
| --- | --- | --- | --- |
| 2009–2013 | 0.205 | NA | NA |
| 2014–2019 | 0.078 | 0.683 | NA |
| 2020+ | 0.016 | 0.108 | 0.201 |

*Pair-wised log-rank p-values.*

**Supplementary figure 9.** Chronologically-stratified cumulative incidence analysis for type 2 endoleaks with all-cause mortality as a competing risk.


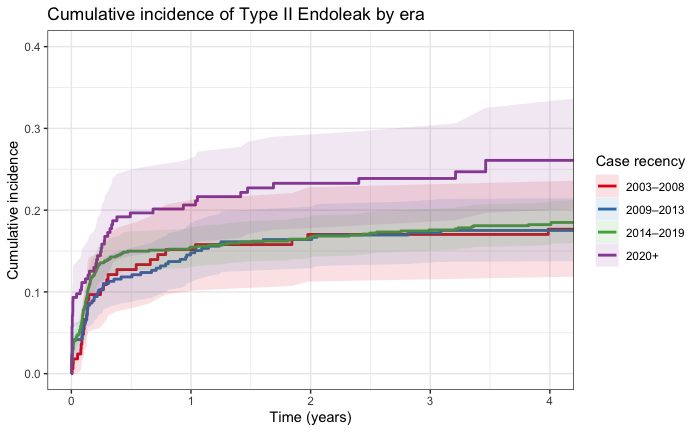


*Gray's test across eras at 4 years, p= 0.047.*

**Supplementary Table 1.** Contributions by collaborating centre.

| **Centre** | **N cases contributed** | **Time period** |
| --- | --- | --- |
| Liverpool | 174 | 2003-2015 |
| St Thomas'/ King’s | 120 | 2007-2018 |
| St Mary's | 186 | 2007-2022 |
| RFH (prev. UCLH) | 33 | 2008-2010 |
| Bristol (prev. Bath) | 175 | 2007-2020 |
| St George's | 231 | 2006-2022 |
| Birmingham | 272 | 2006-2017 |
| Manchester | 17 | 2007-2018 |
| Leicester | 24 | 2007-2010 |
| Cambridge | 81 | 2007-2022 |
| Basildon | 9 | 2018-2022 |
| Southampton | 76 | 2014-2022 |
| Newcastle | 162 | 2007-2022 |
| Norfolk and Norwich | 84 | 2008-2022 |
| Wolverhampton | 7 | 2008-2010 |
| TOTAL | 1651 |  |

**References**

20. van Buuren S, Groothuis-Oudshoorn K. mice: Multivariate Imputation by Chained Equations in R. *J Stat Softw* [Internet]. 2011 [cited 2025 Jul 14];45:1–67. Available from: https://www.jstatsoft.org/index.php/jss/article/view/v045i03

21. Therneau TM. Survival Analysis [R package survival version 3.8-3]. *CRAN: Contributed Packages* [Internet]. 2024 [cited 2025 Jul 8];Available from: https://CRAN.R-project.org/package=survival

22. Kassambara A, Kosinski M, Biecek P. Drawing Survival Curves using “ggplot2” [R package survminer version 0.5.0]. *CRAN: Contributed Packages* [Internet]. 2024 [cited 2025 Jul 8];Available from: https://CRAN.R-project.org/package=survminer

23. Hadley Wickham. ggplot2: Elegant Graphics for Data Analysis. *J R Stat Soc Ser A Stat Soc*. 216AD;174:245–246.

24. Robin X, Turck N, Hainard A, Tiberti N, Lisacek F, Sanchez JC, Müller M. pROC: An open-source package for R and S+ to analyze and compare ROC curves. *BMC Bioinformatics* [Internet]. 2011 [cited 2025 Jul 14];12:1–8. Available from: https://bmcbioinformatics.biomedcentral.com/articles/10.1186/1471-2105-12-77
